# Comprehensive Preclinical Safety Evaluation of Clinical‐Grade hESC‐Derived Multipotent Mesenchymal Stem Cells for Interstitial Cystitis

**DOI:** 10.1155/sci/5539154

**Published:** 2026-08-18

**Authors:** Jeong Min Shin, Ah Reum Kang, Dong Seol Gwak, Su Jin Kim, Jin Won Seo, Eun-Young Kim, Ki-Sung Hong, Se-Pill Park, Hyung-Min Chung

**Affiliations:** ^1^ Department of Research and Development, Mirae Cell Bio Co. Ltd., Seoul, Republic of Korea; ^2^ Department of Bio Medical Informatics, College of Applied Life Sciences, Jeju National University, Jeju, Republic of Korea, jejunu.ac.kr

**Keywords:** biodistribution, human embryonic stem cells, interstitial cystitis, multipotent mesenchymal stem cells, tumorigenicity

## Abstract

Human embryonic stem cell (hESC)‐derived multipotent mesenchymal stem cells (MMSCs) offer therapeutic potential for interstitial cystitis (IC); however, safety concerns, such as tumorigenicity from residual undifferentiated hESCs, must be thoroughly addressed. To address these concerns, we conducted comprehensive in vitro and in vivo safety assessments of clinical‐grade MMSCs. Flow cytometry, qRT‐PCR, and/or RNA sequencing confirmed the absence of pluripotency markers, including *OCT3/4*, *TRA-1-60*, *TRA-1-81*, and *NANOG*, in MMSCs. In good laboratory practice (GLP)‐compliant studies, BALB/c‐*nu/nu* immunodeficient mice received a single intra‐detrusor muscle injection of MMSCs. No adverse clinical signs or immune responses were observed at doses corresponding to up to 6 × 10^9^ cells in humans, the maximum feasible clinical dose based on body weight conversion. Furthermore, no tumor formation was detected for up to 52 weeks following either intra‐detrusor muscle or subcutaneous administration. Spiking studies showed teratoma formation only when undifferentiated hESCs exceeded 1%, supporting the absence of tumorigenic risk in the final MMSC product. Biodistribution analyses revealed that MMSCs remained localized at the injection site and were cleared from major organs within 10 days. These findings indicate that MMSCs contain no detectable hESCs within the sensitivity limits of the assays, exhibit no immunogenicity or tumorigenic potential, and are safe for intra‐detrusor muscle administration, supporting their clinical application as a cell‐based therapy for IC.

## 1. Introduction

Human embryonic stem cells (hESCs) established from the inner cell mass of blastocyst can differentiate into various types of cells in the human body and can be expanded ex vivo as immortalized cell lines [1, 2]. Based on this versatility and infinite scalability, hESCs are considered a promising resource for regenerative medicine [3]. The advantages of hESC derivatives are that they are not limited by donor availability and have excellent scalability.

However, the use of hESCs raises several major concerns. For example, undifferentiated hESCs can form teratomas when transplanted into immunodeficient animals [4]. Furthermore, hESC derivatives may also contribute to tumorigenic risk or provoke an undesirable immune response after transplantation [5]. Nonetheless, hESC derivatives may potentially serve as an unlimited cell source for therapeutic applications provided that safety concerns are adequately addressed. Therefore, stringent quality control and in vivo safety evaluation measures are required to mitigate these risks before clinical application.

We developed an efficient purification procedure for multipotent mesenchymal stem cells (MMSCs) derived from hESCs through epithelial–mesenchymal transition (EMT) using a porous membrane [6]. The MMSCs were expanded and cryopreserved into working cell banks (WCBs) and final products under good manufacturing practice (GMP) conditions. To improve the quality of clinical‐grade banks, we implemented strict quality control measures to test for sterility, viability, identity, purity, potency, and safety of the cells. Previous studies have demonstrated that MMSCs ameliorate bladder dysfunction and attenuate histopathological damage in HCl‐ and LPS‐induced IC models through both differentiation and paracrine effects. MMSCs promote urothelial and stromal regeneration, enhance angiogenesis, and modulate key regenerative pathways, including WNT and Shh, as well as downstream growth factors, such as IGF and FGF. These findings suggest that MMSCs exert therapeutic effects via both cell replacement and microenvironmental modulation [7, 8]. Encouraged by these results, a Phase 1/2a clinical trial to evaluate the safety and efficacy of MMSCs in patients with interstitial cystitis (IC) has been approved by the Ministry of Food and Drug Safety (MFDS) based on a comprehensive set of nonclinical data.

While preclinical studies on MMSCs have demonstrated promising therapeutic potential, systematically reported and comprehensive safety evaluations in the literature remain relatively limited. Therefore, a thorough assessment of the biological safety of MMSCs is essential to support their clinical application and ensure safe administration. In this study, to address safety concerns, we performed qRT‐PCR analysis to detect any residual undifferentiated hESCs in the final products. Additionally, to obtain in vivo safety data, we conducted a comprehensive series of preclinical studies in BALB/c‐*nu*/*nu* mice, including evaluations of general toxicity, tumorigenicity, and biodistribution.

## 2. Materials and Methods

### 2.1. GMP Manufacturing and Quality Testing of MMSCs

GMP manufacturing of MMSCs (code name: MR‐MC‐01), differentiated from hESCs (SNUhES42, passage 13), was performed at the GMP facility of Mirae Cell Bio Co., Ltd., with approval from the Institutional Review Board (IRB; MCB‐001). Donor‐derived SNUhES42 cells were screened for potential infectious agents in accordance with the regulatory guidelines (Table S1). hESC maintenance and differentiation into MMSCs were performed as previously described [6]. MMSCs were expanded in EGM‐MV2 medium (Promocell, Germany) at 37°C in a humidified 5% CO_2_ atmosphere, cryopreserved at passage 7, and used immediately after thawing.

MMSC characterization was confirmed by in vitro differentiation into adipogenic, osteogenic, chondrogenic, and myogenic lineages, following established protocols [9]. For clinical qualification, MMSC banks were evaluated for sterility, mycoplasma, adventitious viral contamination, endotoxin levels, and cellular purity and identity (Table S2).

For GTG‐banding analysis, metaphase arrest was induced by adding colcemid (10 mg/mL). After 3–5 h, the cell monolayer was washed twice with a hypotonic solution (0.075 M KCl containing 1% sodium citrate) and incubated at 37°C for 25 min. The cells were harvested via gentle horizontal shaking and centrifuged at 1500 rpm for 10 min. The cell pellet was fixed with methanol:acetic acid (3:1); this fixation step was repeated twice. The final suspension was dropped onto cold, wet slides and air‐dried. Slides were treated with trypsin and stained with Giemsa (GTG banding). Chromosomes were analyzed under a light microscope at ×1000 magnification.

Genomic stability was further evaluated using high‐resolution array‐based platforms to assess copy number variations (CNVs) and single nucleotide polymorphisms (SNPs). Genomic DNA was extracted using the DNeasy Blood and Tissue Kit (Qiagen, Valencia, CA, USA) according to the manufacturer’s instructions. DNA quality and integrity were assessed using a NanoDrop ND‐2000 UV–Vis spectrophotometer and agarose gel electrophoresis. Only samples exhibiting intact genomic DNA without smearing were used for further analysis. DNA concentrations were quantified using the Quant‐iT PicoGreen reagent (Invitrogen) and adjusted to the required concentrations.

For CNV analysis, genome‐wide profiling was performed using the CytoScan HD array platform (Affymetrix, Santa Clara, CA, USA). Genomic DNA (250 ng) was digested with NspI at 37°C for 2 h, followed by adaptor ligation at 16°C for 3 h. PCR amplification was conducted as follows: 30 cycles of denaturation (94°C, 30 s), annealing (60°C, 45 s), and extension (65°C, 15 s). PCR products were purified, fragmented, and labeled with biotinylated nucleotides using terminal deoxynucleotidyl transferase (37°C, 4 h). Labeled DNA was hybridized to pre‐equilibrated CytoScan HD arrays (50°C, 16–18 h). Array washing and scanning were performed using the GeneChip Scanner platform (Affymetrix). Data were analyzed using the Chromosome Analysis Suite (ChAS) software (human genome build hg38).

For SNP analysis, samples were analyzed using the Infinium Omni5‐4 v1.2_A1 BeadChip platform (Illumina, San Diego, CA, USA). Genomic DNA (200 ng) was subjected to whole‐genome amplification (~1000‐fold). Amplified DNA was fragmented, precipitated with 2‐propanol, and resuspended in a hybridization buffer. DNA samples were denatured (95°C, 20 min) and hybridized to BeadChips (48°C, ≥16 h). Single‐base extension and staining were performed using the Illumina Infinium reagents. BeadChips were scanned using the Illumina iScan Reader, and image data were processed using GenomeStudio software (Illumina).

### 2.2. Flow Cytometric Analysis

Cryopreserved MMSCs were thawed and washed with Perm/Wash buffer (BD Biosciences, USA), followed by surface staining with fluorochrome‐conjugated antibodies against CD44‐PE, CD146‐PE (R&D systems, USA), CD73‐PE, CD90‐PE, CD105‐PE, TRA‐1‐60‐FITC, TRA‐1‐81‐FITC, HLA‐ABC‐APC, HLA‐DR‐PE, CD40‐PE, CD40L‐PE, CD80‐PE, and CD86‐PE (BD Biosciences, USA) for 30 min at 4°C. For intracellular staining, cells were fixed and permeabilized, then stained with Oct3/4 (BD Biosciences, USA). All samples were washed, resuspended in Perm/Wash buffer, and analyzed using a NovoCyte 2000R flow cytometer with NovoExpress software (Agilent, USA).

### 2.3. qRT‐PCR Analysis

RNA was extracted from 1 × 10^6^ cell using the RNeasy Mini Kit (QIAGEN, Germany). cDNA was synthesized from 500 ng of RNA using the Superscript II Reverse Transcriptase (Invitrogen, USA) with oligo(dT)_20_‐primed at 42°C according to the manufacturer’s instructions. The resulting cDNA was diluted 1:2 with nuclease‐free water prior to qRT‐PCR analysis. TaqMan‐based qRT‐PCR was performed in a total reaction volume of 10 μL using a 384‐well plate on an ABI PRISM 7900HT Sequence Detection System (Applied Biosystems, USA). Each reaction contained 5 μL of Universal Master Mix (Applied Biosystems, USA), 90 nM of forward and reverse primers, 250 nM of a fluorescence‐labeled TaqMan probe, and 2 μL of diluted cDNA template. PCR cycling conditions were as follows: initial denaturation at 95°C for 10 min, followed by 40 cycles at 95°C for 15 s and 60°C for 1 min. All samples were analyzed in triplicate, and data were processed using the Sequence Detector software (Applied Biosystems, USA). TaqMan assays specific for *POU5F1* (Hs04260367_gH) and GAPDH (Hs99999905_m1; Thermo Fisher Scientific) were used, and POU5F1 expression levels were normalized to GAPDH.

### 2.4. RNA‐Seq Analysis

We preprocessed the raw reads from the sequencer to remove low‐quality reads and adapter sequences before analysis and aligned the cleaned reads to the *Homo sapiens genome* (*hg19*) using HISAT v2.1.0 [10]. HISAT utilizes two types of indexes for alignment: a global whole‐genome index and tens of thousands of small local indexes. These two types of indexes are constructed using the same Burrows–Wheeler Transform (BWT) and a graph FM index (GFM) as used in Bowtie2. Due to its utilization of these efficient data structures and algorithms, HISAT generates spliced alignments several times faster than Bowtie and BWA widely used. The reference genome sequence for *Homo sapiens genome* (*hg19*) and annotation data were obtained from the NCBI. Subsequently, transcript assembly and quantification were performed using StringTie v1.3.4b [11, 12]. Based on the results, the expression abundance of transcripts and genes was calculated as read counts or FPKM value (Fragments Per Kilobase of exon per million fragments mapped) per sample. The expression profiles were utilized for additional analyses, such as differentially expressed genes (DEGs). When comparing groups with different conditions, DEGs or transcripts were filtered using statistical hypothesis testing.

### 2.5. ELISA for the Detection of 5‐Bromo‐2^′^‐Deoxyuridine (BrdU) Incorporation and Interferon‐Gamma (IFN‐γ) Secretion

For the immunogenicity assay, the absence of immunostimulatory activity of MMSCs was evaluated. Cell proliferation was assessed using the Cell Proliferation ELISA Kit (Roche, Switzerland) based on BrdU incorporation in accordance with the manufacturer’s instructions. Mixed lymphocyte reaction (MLR) assays were conducted in 96‐well plates using 0.2 mL of modified RPMI 1640 medium (Gibco, USA) supplemented with 10% heat‐inactivated fetal bovine serum. A total of 1 × 10^5^ peripheral blood mononuclear cells (PBMCs) from an allogeneic donor (responder) were cultured for 6 days either alone or co‐cultured with the following:

• 1 × 10^5^ mitomycin C (MMC)‐treated PBMCs from a second allogeneic donor (stimulator), used as a positive control.
• Phytohemagglutinin‐L (PHA, 1 μg/mL), used as a positive control.
• 1 × 10^5^ MMC‐treated MMSCs.

Additionally, MMC‐treated stimulator PBMCs and MMC‐treated MMSCs were also cultured separately at a density of 1 × 10^5^ cells per well. BrdU was added to each group, and cell proliferation was assessed by measuring the optical density corresponding to BrdU incorporation. The proliferation index (%) was calculated using the following formula: [(OD_responder PBMCs + MMSCs_) – (OD _MMSCs_) / (OD_responder PBMCs + stimulator PBMCs_) – (OD_stimulator PBMCs_)] × 100 [13].

Following the same culture conditions used for the BrdU incorporation assay, each group was incubated for 6 days. After incubation, the culture supernatants were carefully collected and analyzed for IFN‐γ concentrations using a commercially available IFN‐γ ELISA kit (BioLegend, USA) according to the manufacturer’s instructions.

### 2.6. Animal Experiments

A series of safety and toxicity studies for MMSCs were performed using BALB/c‐*nu*/*nu* mice at CorestemChemon Inc., a good laboratory practice (GLP)‐certified contract research organization (CRO). The BALB/c‐*nu/nu* mice used in this study are inbred animals characterized by generalized alopecia and the absence of a thymus and thymus‐derived lymphocytes. Owing to their immunodeficient phenotype, they are widely used as animal models in immunological research, including tumor implantation, cell transplantation, and tumorigenicity studies. For all preclinical studies, mice were housed in polypropylene cages under controlled conditions (23 ± 3°C, 12‐h light/dark cycle, 55 ± 15% relative humidity) with ad libitum access to food and water and acclimated for 7 days prior to study initiation. The body weights of animals deemed healthy during the acclimation period were measured and ranked. Healthy animals with body weights proximal to the group mean were selected and randomly assigned to experimental groups, as summarized in the group allocation tables. To minimize observer bias, the personnel responsible for clinical observations and pathological evaluations were blinded to group allocation.

For the general toxicity study, mice were divided into four treatment groups (10 animals per sex per group). Group sizes were determined in accordance with regulatory guidelines for GLP‐compliant general toxicity studies. MMSCs or vehicle were administered via the intra‐detrusor muscle route (0.05 mL/site, two sites per animal) at doses of 8 × 10^4^, 4 × 10^5^, or 2 × 10^6^ cells/mouse (Table S3). The primary objectives were to identify potential dose‐limiting toxicities (DLTs) and to determine the no observed adverse effect level (NOAEL). Safety assessments included monitoring of clinical signs, body weight, food consumption, ophthalmological examinations, urinalysis, hematology, clinical biochemistry, organ weights, and gross necropsy findings. Additionally, lymphocyte phenotype analysis was conducted to assess the potential immunotoxicity. Animals were observed daily for 13 weeks, after which surviving mice were euthanized via CO_2_ and subjected to necropsy. Immune responses were further evaluated by flow cytometric analysis of splenic immune cell subsets using antibodies against CD3‐Cy7, CD4‐PE, CD8‐FITC, CD45R/B220‐FITC, and CD49b‐PE (with appropriate isotype control) on a BD Accuri C6 flow cytometry (BD Biosciences, USA).

The tumorigenicity study was conducted in 10‐week‐old BALB/c‐*nu*/*nu* mice. Group sizes (10 animals per sex per group) were determined in accordance with WHO recommendations and regulatory guidance for the tumorigenicity assessment of cell‐based products. The primary endpoint was tumor incidence, confirmed via gross and histopathological examination. MMSCs were administered either subcutaneously (1 × 10^7^ cells per mouse) or via intra‐detrusor muscle injection (2 × 10^6^ cells per mouse). For subcutaneous administration, cells were suspended in 0.2 mL of vehicle (normal saline), whereas intra‐detrusor muscle injection were performed in 0.1 mL per mouse. HeLa cells (ATCC, CCL‐2) were used as a positive control, in compliance with WHO guidelines [14] (Table S4). Mice were monitored for mortality, clinical signs, body weight, food consumption, and ophthalmological findings over a 52‐week period. Tumor formation was assessed daily via palpation and visual inspection. Animals exhibiting measurable tumors were euthanized for immediate histopathological evaluation. All surviving mice underwent necropsy and histopathological examination at study termination.

To further evaluate tumorigenic potential, MMSCs spiked with their parental hESC line (SNUhES42) were administered subcutaneously or via intra‐detrusor muscle injection using saline and a Matrigel (Corning, USA) mixture, in accordance with MFDS recommendations. A total of 2 × 10^6^ cells per mouse were delivered with varying proportions of hESCs (1%, 2%, or 100%) (Table S5). Animals (15 animals/sex/group) were monitored for 52 weeks and euthanized upon tumor detection or at study termination for necropsy and histopathological analysis.

The in vivo biodistribution kinetics of MMSCs were evaluated in immunodeficient BALB/c‐*nu*/*nu* mice (30 animals/sex) following intra‐detrusor muscle administration at a dose of 2 × 10^6^ cells per mouse. The number of animals per time point (*n* = 3 per sex) was determined in accordance with regulatory recommendations for the biodistribution assessment of cell therapy products. The primary endpoint was the detection and persistence of human *Alu* sequences in harvested organs. At multiple time points up to 52 weeks, animals (*n* = 3 per sex) were euthanized for organ collection and subsequent analysis (Table S6).

Biodistribution and persistence were assessed via qRT‐PCR targeting the human *Alu* sequence in multiple organs, including the brain, spinal cord, blood, heart, lung, liver, spleen, kidney, mesenteric lymph nodes, mandibular lymph node, bone marrow, pancreas, epididymis, urinary bladder, ovary, testis, and uterus. No predefined exclusion criteria were established prior to study initiation. No animals or data points were excluded unless death occurred prior to scheduled termination, in which case all available data were included. Animals were euthanized in accordance with the Institutional Animal Care and Use Committee (IACUC) guidelines of the CorestemChemon Nonclinical Research Center if clinical signs of severe distress were observed. Moribund or spontaneously deceased animals were subjected to necropsy procedures equivalent to those performed for scheduled terminations. Animal experiments were approved by the IACUC under specific protocols: [18‐M558] for single‐dose toxicity, [18‐M671] for tumorigenicity, [2023‐0480] for hESC spiking, and [18‐M803] for biodistribution.

### 2.7. Statistical Analysis

All experimental data from qRT‐PCR, ELISA, and safety and toxicity studies were expressed as the mean ± standard deviation (SD). Data obtained from the biodistribution study were expressed as the mean ± standard error (SE). Statistical analyses were performed using SPSS Statistics version 22 (IBM SPSS Statistics, Armonk, NY, USA). To assess the assumption of homogeneity of variance, Levene’s test was used. When the assumption of homogeneity of variance was satisfied, one‐way ANOVA was performed, followed by the Dunnett’s test for post‐hoc comparisons between the control and treatment groups. When the assumption of homogeneity of variance was not satisfied, Welch’s ANOVA was performed, followed by the Dunnett’s T3 test. Significance was considered as  ^∗^ *p* < 0.05,  ^∗∗^ *p* < 0.01, and  ^∗∗∗^ *p* < 0.001.

## 3. Results

### 3.1. Characterization of GMP‐Produced MMSCs

**Figure 1:**
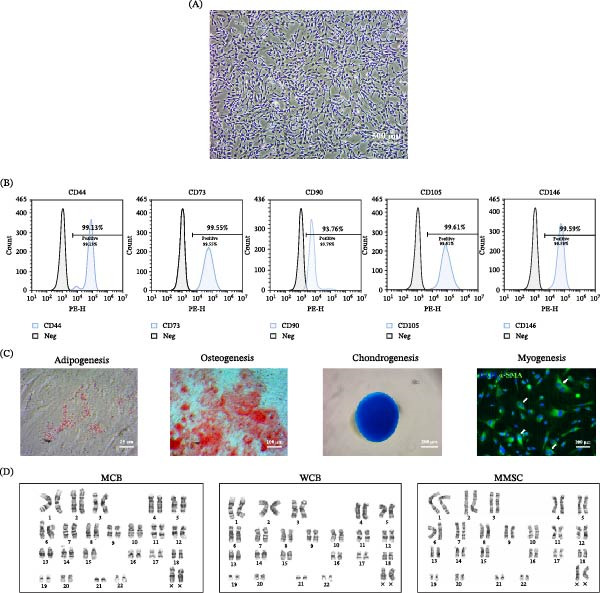
Characterization of MMSCs. (A) Representative phase‐contrast image showing the fibroblastic morphology of MMSCs. Scale bar = 500 μm. (B) Flow cytometric quantification of MSC markers (CD44, CD73, CD105, and CD146). (C) Assessment of multilineage differentiation capability of MMSCs (adipogenic, osteogenic, chondrogenic, and myogenic differentiation). Arrows indicate multinucleated cells. Scale bars = 25 μm (adipogenesis), 100 μm (osteogenesis), 200 μm (chondrogenesis), and 100 μm (myogenesis). (D) Representative GTG‐banded karyotypes of the Master Cell Bank (MCB), Working Cell Bank (WCB), and final MMSC products.

To characterize the cellular identity of the MMSCs, we first examined their surface marker expression profile and multilineage differentiation potential. MMSCs consistently exhibited a spindle‐shaped fibroblast‐like morphology (Figure 1A). Flow cytometry analysis revealed that MMSCs expressed typical mesenchymal stem cell (MSC) markers, including CD44, CD73, CD90, CD105, and CD146 (Figure 1B). These expression patterns were consistently observed across independent MMSC preparations, indicating phenotypic stability. Furthermore, MMSCs demonstrated robust multilineage differentiation capacity under lineage‐specific induction conditions. Following adipogenic differentiation, intracellular lipid droplet formation was evident, whereas osteogenic differentiation resulted in marked matrix mineralization. Chondrogenic differentiation led to the formation of a proteoglycan‐rich extracellular matrix (ECM), and myogenic differentiation was confirmed by positive immunofluorescent staining for α‐smooth muscle actin (α‐SMA) (Figure 1C). Collectively, these findings demonstrate that MMSCs satisfy the phenotypic and functional criteria for MSCs as defined by the International Society for Cell & Gene Therapy (ISCT), confirming their multipotent characteristics. Quantitative evaluation across three independent clinical‐grade MMSC lots further demonstrated high batch‐to‐batch consistency in critical quality attributes, including cell viability (mean 94.50 ± 1.06%) and surface marker expression profile.

The genomic stability of MMSCs during in vitro expansion and differentiation was evaluated via GTG‐banding karyotype analysis, genome‐wide CNV analysis with the CytoScan HD array, and SNP genotyping with the Infinium Omni5‐4 v1.2 platform. Karyotype analysis of the Master Cell Bank (MCB), the WCB, and the final MMSC products (up to passage 7) consistently demonstrated a normal diploid chromosomal complement with no clonal numerical or structural abnormalities (Figure 1D). Genome‐wide CNV analysis of the final MMSC products (passage 7) revealed no detectable copy number gains or losses (gain = 0; loss = 0) after filtering based on predefined criteria (segment size ≥25 kb, marker count ≥10, and exclusion of variants reported in the Database of Genomic Variants and cancer‐associated regions). Furthermore, SNP genotyping comparing the WCB (passage 3) and final MMSC products (passage 7) showed consistent genomic profiles, with no evidence of CNV or loss of heterozygosity (LOH) (Figure S1). Collectively, these complementary analyses indicate that no detectable genomic alterations were introduced during cell expansion or the EMT process, thereby confirming the genomic stability of the MMSCs throughout the manufacturing process within the resolution limits of the assays utilized.

### 3.2. Clinical‐Grade MMSCs Are Free of Detectable Residual Pluripotent hESCs

**Figure 2:**
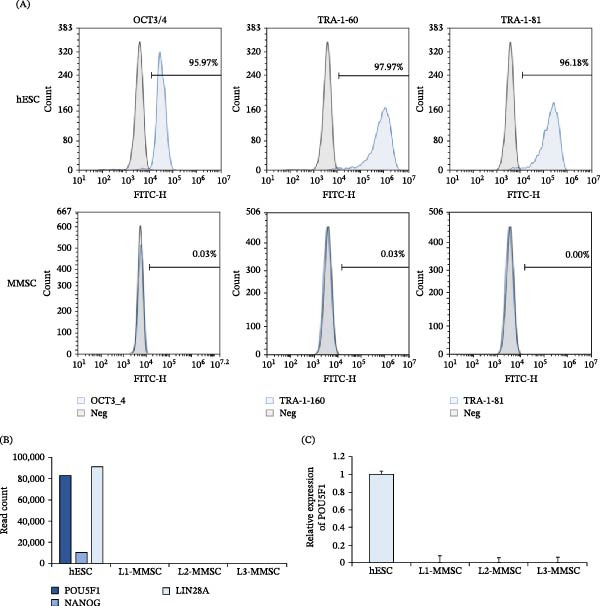
Cell purity assessment of MMSCs. (A) Representative flow cytometry analysis of undifferentiated hESCs and MMSCs for the pluripotency markers OCT3/4, TRA‐1‐60, and TRA‐1‐81. (B) Analysis of pluripotency gene expression levels via RNA‐seq in hESCs and three batches of MMSCs. (C) qRT‐PCR analysis of POU5F1 gene expression level in hESCs and three batches of MMSCs. L1‐MMSC, Lot 1‐MMSC; L2‐MMSC, Lot 2‐MMSC; L3‐MMSC, Lot 3‐MMSC. The data are presented as the mean ± SD of three independent experiments.

The potential persistence of undifferentiated hESCs poses a significant safety concern due to their tumorigenic potential. To rigorously evaluate this risk, we conducted a multi‐tiered analytical approach to determine whether any residual hESCs remained within the clinical‐grade MMSC population. First, flow cytometry analysis was performed to assess the expression of pluripotency markers (Oct3/4, Tra‐1‐60, and Tra‐1‐81), using undifferentiated hESCs as a positive control. MMSCs were negative for all tested markers, confirming their absence within the sensitivity limits of the assay (Figure 2A).

To further evaluate pluripotency at the transcript level, RNA‐seq was employed as a global screening tool to assess gene expression profiles. RNA‐seq analysis did not detect the expression of key pluripotency‐associated genes, including *POU5F1*, *NANOG*, and *LIN28A*, within the sensitivity limits of the method (Figure 2B). Subsequently, qRT‐PCR targeting *POU5F1* (*Oct3/4*), a representative and highly specific marker of pluripotency [15, 16], was performed for the sensitive detection of residual hESC‐associated transcripts. The sensitivity of the POU5F1 qRT‐PCR assay was evaluated using hESC‐spiked MMSC samples at concentrations ranging from 100% to 0.01%, establishing a limit of detection (LOD) of 0.23% of POU5F1 mRNA (data not shown). Consistent with the RNA‐seq results, *POU5F1* expression was not detected in the final MMSC products, whereas robust expression was observed in hESC positive controls (Figure 2C).

Collectively, these results indicate that there are no detectable residual hESCs within the sensitivity limits of the applied assays, with qRT‐PCR providing the primary quantitative validation of the MMSC purity profile.

### 3.3. Absence of Immunogenicity in MMSCs

**Figure 3:**
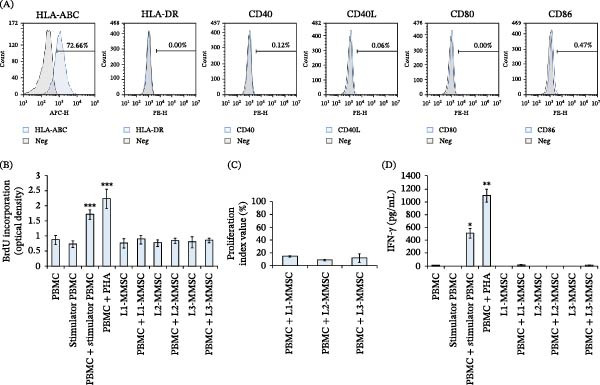
Immunogenicity assessment of MMSCs. (A) Quantitation of immunological property markers (HLA‐ABC, HLA‐DR, CD40, CD40L, CD80, and CD86) using flow cytometry analysis. (B) BrdU assay revealing that optical density in PBMCs co‐cultured with MMSCs in comparison with control MLRs containing PBMC plus stimulator PBMC. (C) Proliferation index value of MMSCs compared to stimulator PBMCs in MLR assay. (D) Measurement of secreted IFN‐γ levels in MLR assay. L1‐MMSC, Lot 1‐MMSC; L2‐MMSC, Lot 2‐MMSC; L3‐MMSC, Lot 3‐MMSC. The data are presented as the mean ± SD of three independent experiments.

To evaluate the immunogenicity of the MMSCs, we performed flow cytometric analysis to assess the expression of MHC class II (HLA‐DR) and key co‐stimulatory molecules (CD40, CD40L, CD80, and CD86). Analysis revealed that MMSCs lacked detectable expression of HLA‐DR and these co‐stimulatory molecules (Figure 3A), suggesting an immune‐evasive phenotype based on their surface marker profiles.

To further rigorously assess the immunogenicity of MMSCs, a MLR assay was conducted using PBMCs [17–19]. PBMCs were co‐cultured with MMSCs, allogeneic stimulator PBMCs (positive control), or phytohemagglutine (PHA), and PBMC proliferation was quantified via the BrdU incorporation assay. Compared with the negative control (donor PBMCs, optical density [OD] = 0.86 ± 0.15), PBMCs co‐cultured with each of the three MMSC lots (L1, L2, or L3) exhibited comparable OD values of 0.87 ± 0.14, 0.83 ± 0.08, and 0.84 ± 0.07, respectively. In contrast, the positive control groups (stimulator PBMCs) showed significantly elevated OD values (1.70 ± 0.16), and treatment with PHA induced a robust proliferative response (OD = 2.23 ± 0.32), validating the assay sensitivity (Figure 3B). These results confirm that MMSC co‐culture does not elicit a proliferative response in PBMCs, with all values remaining well within the predefined acceptance criterion (proliferation index ≤ 54%) [20] (Figure 3C).

In addition to cell proliferation, we examined whether MMSC interaction influenced the secretion of the pro‐inflammatory cytokine IFN‐γ. We measured IFN‐γ levels in the MLR supernatant and compared them with those of PBMCs cultured with the allogeneic stimulator PBMCs. While co‐culture with stimulator PBMCs resulted in markedly elevated IFN‐γ levels (508.40 ± 74.34 pg/mL) as compared to their basal levels (2.39 ± 0.52 pg/mL). PBMCs co‐cultured with the three MMSC lots exhibited minimal IFN‐γ secretion, with values comparable to basal levels (11.99 ± 5.30, 0.23 ± 0.28, and 4.51 ± 4.80 pg/mL) (Figure 3D). These results indicate that MMSCs did not induce an immune response, as evidenced by the absence of IFN‐γ upregulation in the co‐culture conditions.

### 3.4. General Toxicity Study of MMSCs Following Single Intra‐Detrusor Muscle Dose Administration

To evaluate the general toxicity of MMSCs following administration, a 13‐week toxicity study was conducted in immunodeficient BALB/c‐*nu*/*nu* mice. Animals received intra‐detrusor injections of MMSCs at varying doses and were closely monitored for safety parameters throughout the study. No treatment‐related mortality or clinical signs were observed. Furthermore, no local adverse reactions noted at the injection sites in any treatment group. Although two male mice (one from the low‐dose group and one from the vehicle control group) died on days 32 and 83, respectively, these events were not considered related to MMSC administration as no corresponding pathological or histopathological abnormalities were identified (data not shown).

Clinical biochemistry parameters were analyzed to assess systemic toxicity. The majority of parameters showed no significant differences. Minor fluctuations that reached statistical significance were observed only in the high‐dose female group (2 × 10^6^ cells per animal) for the albumin/globulin (A/G) ratio (*p* = 0.027) and Na+ levels (*p* = 0.0013) compared with the vehicle control. However, these changes were considered toxicologically irrelevant because the absolute values remained within the normal physiological reference ranges, lacked a clear dose‐dependent trend, and were not accompanied by any associated histopathological abnormalities (Table S7). Based on these findings, the NOAEL for MMSCs following a single intra‐detrusor muscle administration in mice was determined to be 2 × 10^6^ cells per animal.

This NOAEL of 2 × 10^6^ cells in 20 g mouse corresponds to a human equivalent dose (HED) of ~6 × 10^9^ cells for 60 kg human [21]. Our clinical dose of 2 × 10^7^ cells per 60 kg human provides a safety margin exceeding 300‐fold relative to the NOAEL, demonstrating a robust safety profile.

To further evaluate potential immunotoxicity, immune cell subset analysis was performed on spleen samples collected at 13 weeks post‐injection. No statistically significant differences were observed in the proportions of CD3^+^ T cells, CD3^+^CD4^+^ helper T cells, CD3^+^CD8^+^ cytotoxic T cells, CD45R^+^B220^+^ B cells, or CD49b^+^ natural killer (NK) cells between MMSC‐treated groups and the vehicle control group (Table 1, all *p* > 0.05). These results demonstrate that MMSC administration did not alter splenic lymphocyte composition, indicating that a single administration of MMSCs does not elicit an immune response in this model.

**Table 1:** Splenic immune cell subsets of MMSCs‐administered mice.

Sex	Dose (cells/head)	Percentage (%)				
		CD3+ T cells	CD3+ CD8+ Tc cells	CD3+ CD4+ Th cells	CD45R+ B220+ B cells	CD49b+ NK cells
Male	0	5.36 ± 2.04	0.54 ± 0.51	1.76 ± 1.18	86.96 ± 3.67	5.98 ± 2.51
	8 × 10^4^	4.96 ± 0.88	0.26 ± 0.15	1.46 ± 0.76	87.02 ± 3.60	7.50 ± 3.98
	4 × 10^5^	4.98 ± 0.76	0.32 ± 0.33	1.78 ± 0.40	86.04 ± 3.65	7.06 ± 3.05
	2 × 10^6^	4.60 ± 0.73	0.22 ± 0.08	1.22 ± 0.86	88.06 ± 2.30	7.10 ± 2.54
Female	0	6.26 ± 1.40	0.66 ± 0.31	2.02 ± 0.75	84.50 ± 3.26	9.62 ± 2.04
	8 × 10^4^	6.24 ± 1.23	0.62 ± 0.40	1.94 ± 0.62	84.22 ± 3.16	10.50 ± 2.57
	4 × 10^5^	8.00 ± 3.64	0.96 ± 1.12	3.06 ± 1.72	86.24 ± 1.24	8.50 ± 2.39
	2 × 10^6^	4.88 ± 0.44	0.46 ± 0.18	1.04 ± 0.53	84.78 ± 2.41	10.16 ± 1.15

*Note:* Data are presented as the mean ± SD.

Abbreviations: NK cells, natural killer cells; T cells, total T cells; Tc cells, cytotoxic T cells; Th cells, helper T cells.

### 3.5. Absence of Tumorigenic Potential in MMSCs

A 52‐week tumorigenicity study was conducted in immunodeficient BALB/*c-nu/nu* mice to assess the tumorigenic risk of clinical‐grade MMSCs. The experimental design included several treatment groups, with HeLa cells being administered as a positive control to validate the sensitivity of the assay system. Mice were monitored for tumor formation, abnormal tissue growth, and systemic adverse effects throughout the study period.

**Figure 4:**
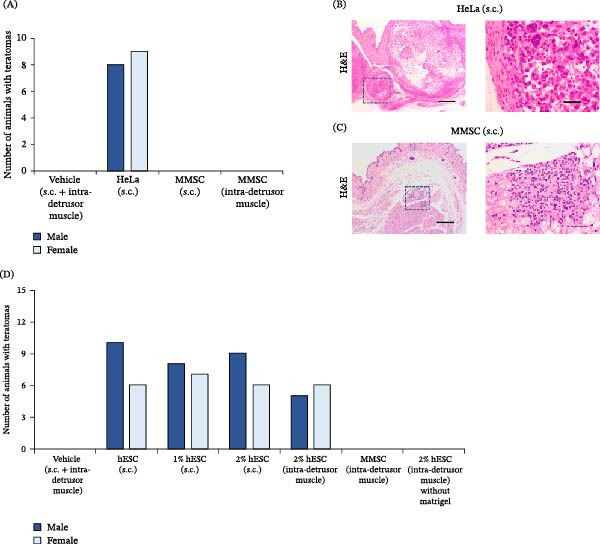
Tumorigenicity and hESC spiking assessment of MMSCs. (A) Number of animals with teratomas in each group: vehicle (negative control), HeLa cells (positive control), and MMSCs (test article). (B, C) Histopathological evaluation of local reactions in the subcutaneous tissue of BALB/*c-nu*/*nu* mice following injection of 200 µL containing HeLa cells or MMSCs (1 × 10^7^ cells/mouse). In the HeLa‐injected mouse, H&E staining revealed carcinoma structures (dotted square). Scale bars, 3000 μm (left) and 100 μm (right). In the MMSC‐injected mouse, representative images showed infiltrated granulomatous lesions (dotted square). Scale bars, 1000 μm (left) and 100 μm (right). (D) Frequency of teratoma formation in mice injected with MMSCs or MMSCs spiked with increasing levels of hESCs.

As expected, most mice in the positive control group (HeLa cells) experienced early mortality or required euthanasia due to tumor progression; teratomas were confirmed in 8 of 10 males and 9 of 10 females, validating the sensitivity of the tumorigenicity assay (Figure 4A). Histological analysis via H&E staining revealed characteristic malignant carcinoma structures in the subcutaneous tissues of these mice (Figure 4B). In contrast, no tumors or abnormal neoplastic growths were detected in mice administered with MMSCs via the intra‐detrusor muscle injection. While localized nodules were observed in the subcutaneous MMSC‐treated group, histopathological examination revealed these to be clusters of cells surrounded by granulomatous inflammatory tissue, with no atypical cellular features, malignant transformation, or evidence of vascular invasion (Figure 4C).

No treatment‐related clinical signs or adverse findings were observed in any MMSC‐treated group. Furthermore, no statistically significant differences were observed in comprehensive safety parameters, including body weight, food intake, ophthalmic findings, blood analyses, organ weights, necropsy, or histopathology, when compared with the vehicle‐control group. Collectively, these results demonstrate that clinical‐grade MMSCs did not induce tumor formation or any adverse outcomes in immunodeficient mice throughout the 52‐week observation period. This long‐term in vivo tumorigenicity assessment provides robust evidence supporting the safety of MMSCs for clinical application.

### 3.6. Evaluation of Teratoma Formation Following hESC Spiking in MMSCs

To evaluate the tumorigenic potential of potential residual hESCs, a spiking study was conducted in which MMSCs were mixed with various proportions of undifferentiated hESCs and administered to immunodeficient mice [22]. As expected, no teratomas were observed in mice injected subcutaneously with the vehicle control, whereas teratomas formed in a substantial proportion of mice injected with 100% hESCs mixed with Matrigel (66.7% of males [10/15] and 40.0% of females [6/15]). Teratomas were also detected at varying frequencies in groups spiked with 1 or 2% hESCs. In the 1% hESCs‐spiked group, teratomas developed in 53.3% of males (8/15) and 46.7 of females (7/15), while in the 2% hESCs‐spiked group, the incidence was 60.0% (9/15) in males and 40.0% (6/15) in females. Teratoma formation was consistently observed in all groups containing hESCs, regardless of the spiking proportion.

Furthermore, when 2% hESC‐spiked MMSCs were administered via the intra‐detrusor muscle, the intended clinical delivery route, teratomas were observed in 33.3% of males (5/15) and 40.0% of females (6/15). In contrast, no teratomas were observed in any animals injected with 100% MMSCs via the intra‐detrusor muscle route (Figure 4D). Teratomas identified at the injection sites (subcutaneous and intra‐detrusor) in the 1% or 2% hESCs‐spiked groups were histopathologically confirmed as benign tumors comprising tissues from all three germ layers, including epidermal cells, chondrocytes, mucous cells, adipocytes, and bone tissue. These teratomas exhibited histopathological features consistent with differentiation into endodermal, mesodermal, and ectodermal lineages, similar to those observed in the positive control group (100% hESCs) (data not shown). These findings validate the tumorigenic potential of residual hESCs and confirm the absence of such cells in clinical‐grade MMSCs within the sensitivity limits of our detection assays.

### 3.7. Biodistribution Study of MMSCs

The biodistribution of MMSCs was assessed in various tissues collected at 12 h; 1, 3, and 10 days; and 4, 8, 12, 26, and 52 weeks post‐administration via qRT‐PCR targeting human‐specific *Alu* repeat sequences (Table S8). The panel of collected tissues included the brain, liver, blood, lungs with mainstem bronchi, bone marrow, mandibular lymph nodes, mesenteric lymph nodes, urinary bladder (injection site), spinal cord, spleen, heart, kidneys, testes, epididymis, ovaries, and uterus. The linearity, accuracy, precision, and specificity of the qRT‐PCR assay were validated prior to study initiation (data not shown).

In the vehicle‐control group, no human *Alu* sequences were detected in any organ or tissue. In MMSCs‐treated male mice, human *Alu* gene expression was detected exclusively in the urinary bladder tissue at 12 h, 1 day, and 3 days post‐injection, with detected levels decreasing over time (1.847 × 10^5^, 0.332 × 10^5^, and 0.149 × 10^5^ cells per urinary bladders, respectively). Similarly, in female mice, human *Alu* sequences were detected only in the urinary bladder at the same time points and exhibited a time‐dependent decline (1.650 × 10^5^, 0.319 × 10^5^, and 0.075 × 10^5^ cells per urinary bladder, respectivelyFigure 5; and Table S9).

**Figure 5:**
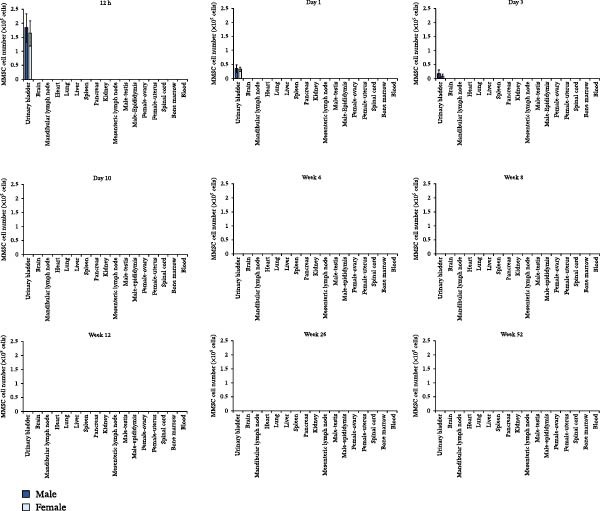
Biodistribution assessment of MMSCs at 12 h, 1, 3, and 10 days and 4, 8, 12, 26, and 52 weeks after administration inside the bladder tissue of BALB/c‐*nu*/*nu* mice. After injecting 2 × 10^6^ MMSCs into the intra‐detrusor muscle of BALB/*c-nu*/*nu* mice, qRT‐PCR analysis was conducted to assess human *Alu* gene expression in different organs. The data are presented as the mean ± SE of three independent experiments.

Human gene expression was not detected in any other tissues in either sex. Furthermore, by day 10, all organs, including the injection site, tested negative for human DNA (Figure 5). Collectively, these findings demonstrate that MMSCs remain localized at the injection site (urinary bladder) and do not migrate to distant organs. The transplanted cells undergo gradual clearance, becoming undetectable in all analyzed tissues—including the injection site and reproductive organs—by day 10 post‐administration.

## 4. Discussion

As MMSCs are derived from hESCs, the potential presence of residual undifferentiated cells poses a significant safety concern, particularly regarding tumorigenicity. In this context, we conducted a comprehensive series of in vitro and in vivo studies to evaluate the safety profile of clinical‐grade MMSCs, focusing on their toxicity and tumorigenic potential.

To specifically assess the presence of residual pluripotent cells, we employed multiple molecular and cellular assays targeting key pluripotency markers. Flow cytometry for Oct3/4, TRA‐1‐60, and TRA‐1‐81, along with RNA‐seq analysis for POU5F1, NANOG, and LIN28A, confirmed the absence of these markers. Furthermore, qRT‐PCR demonstrated that the POU5F1 expression was nearly undetectable. Collectively, these independent assays indicate that clinical‐grade MMSCs contain no detectable residual hESCs within the sensitivity limits of the assays, thereby minimizing the risk associated with pluripotent cell contamination.

Genomic stability was evaluated using GTG‐banding karyotype analysis and high‐resolution array‐based methods, including CNV analysis and SNP genotyping. MMSCs from the MCB, the WCB, and the final product (up to passage 7) exhibited normal diploid karyotypes with no clonal chromosomal abnormalities. Furthermore, no CNVs or LOH was detected across the genome, and no genomic differences were observed between early and late passages. These results indicate that neither in vitro expansion nor the EMT process introduced detectable genomic alterations, confirming the genomic stability of MMSCs within the resolution limits of the applied methods.

We further assessed the immunogenicity and toxicity of MMSCs. The absence of MHC class II and co‐stimulatory molecules such as CD40, CD40L, CD80, and CD86 suggests an immune‐evasive phenotype (Figure 3A). While the possibility remains that allogeneic stem cell therapeutics derived from donors carrying genetic variations may exhibit variable expression of these molecules. In this study, we evaluated the general toxicity of MMSCs, including their potential immunotoxicity. Although Morizane et al. previously reported that alloimmune responses against graft‐derived antigens can lead to clinical symptoms and graft rejection [23], our findings demonstrated that MMSCs did not induce any signs of immune rejection or adverse clinical effects. Furthermore, the MLR assay showed that MMSCs did not induce PBMC proliferation or activate immune cells, indicating low immunogenicity.

Based on the NOAEL of 2 × 10^6^ cells established in our toxicity study, we evaluated the long‐term in vivo safety of MMSCs, focusing on tumorigenicity and biodistribution over 52 weeks. HeLa cells, used as a positive control, validated the sensitivity of our tumorigenicity assay. Notably, no teratoma formation was observed in animals administered MMSCs via either the subcutaneous or intra‐detrusor muscle route, indicating that MMSCs lack tumorigenic potential under the clinically relevant conditions.

To further address the risk of residual hESCs, we conducted a spiking study. In the initial test, MMSCs spiked with hESCs were suspended in saline and injected subcutaneously into mice. However, the spiked hESCs did not form teratomas (data not shown). Based on these results, we suggest that the absence of a supportive microenvironment limited the tumorigenicity of the spiked hESCs. Although teratomas can theoretically arise from a single undifferentiated cell, a threshold level is required, as demonstrated in previous spiking studies [22, 24–27]. To investigate the contribution of the local microenvironment, we performed supplementary experiments using Matrigel, which provides ECM components and pro‐angiogenic factors that promote cell survival, engraftment, and proliferation [22, 28, 29]. In this context, the Matrigel‐based condition may represent a highly permissive or “worst‐case” scenario for tumor formation. In contrast, the physiological environment of the detrusor muscle lacks such exogenous matrix support and is relatively less permissive to the survival and expansion of residual undifferentiated cells [30, 31].

Subcutaneous injection of 1% and 2% hESCs (~20,000 and 40,000 cells, respectively) resulted in teratoma formation. Similarly, injection of 2% hESCs into the detrusor muscle also led to teratoma development. In contrast, injection of 2% hESCs without Matrigel into the detrusor muscle did not result in teratoma formation. This contrast strongly implies that the tumorigenic potential of undifferentiated cells is significantly influenced by the presence of angiogenic and matrix‐supporting factors provided by Matrigel [32].

These findings indicate that the tumorigenic risk is not solely determined by the presence of pluripotent cells but is also strongly influenced by the local tissue microenvironment. Importantly, the absence of tumor formation following administration of MMSCs in the presence of Matrigel—a permissive environment for teratoma development—suggests that any residual pluripotent cells were present at levels below the threshold required for tumor formation.

Apart from teratoma formation, sporadic occurrences of lymphomas, pulmonary adenomas, histiocytic sarcomas, hemangiomas, polyps, and leukemias were observed at distant sites from the injection area (Table S10). However, similar incidences were also detected in the vehicle control group, suggesting that these findings are incidental or spontaneous rather than related to the test substance [33–35].

Collectively, these results provide strong preclinical evidence for the in vivo safety of MMSCs, with no tumorigenicity observed even at the highest feasible dose under clinically relevant conditions. The inclusion of appropriate positive controls and a Matrigel‐dependent spiking model further supports the validity and sensitivity of the experimental system, while also highlighting the critical role of the microenvironment in modulating the tumorigenic behavior of stem cell‐based therapeutics.

We also evaluated the biodistribution of MMSCs following intra‐detrusor muscle injection. The administered cells remained localized at the injection site and did not migrate to distant organs, including reproductive organs. Cell concentrations at the injection site decreased within a short period, reaching undetectable levels by day 10. These findings indicate that intra‐detrusor administration does not result in systemic distribution or long‐term engraftment, further supporting the local safety profile of the product.

Importantly, the observed transient persistence of MMSCs contradicts their previously reported long‐term therapeutic efficacy in IC models. In our earlier studies [7, 8], transplanted MMSCs remained detectable at the injection site for extended durations (up to 181 and 30 days in HCl‐ and LPS‐induced IC animal models, respectively), indicating that cell persistence is highly context‐dependent. This discrepancy is likely attributable to the differences in the host microenvironment. In immunodeficient mice used for biodistribution studies, the absence of injury‐associated signals and the presence of activated innate immune components (e.g., NK cells in BALB/c‐nu/nu mice) may contribute to the rapid clearance of transplanted cells [36–40]. In contrast, tissue injury involves dynamic phases of inflammation, repair, and remodeling, during which immune responses are actively modulated [41]. It has been reported that the therapeutic efficacy of MSCs is highly dependent on the inflammatory context, with stronger therapeutic effects observed under conditions of pronounced inflammation, whereas limited efficacy is seen in low‐inflammatory environments [42–44]. Within this context, inflamed and injured bladder tissues in IC animal models provide a pro‐regenerative microenvironment that supports cell survival, engraftment, and functional activity. Importantly, the therapeutic effects of MMSCs are considered to be mediated not only by direct cell engraftment but also, to a large extent, through paracrine mechanisms. MMSCs contribute to bladder repair by supporting angiogenesis in urothelial, stromal, and perivascular compartments, while secreting a broad range of cytokines and growth factors that promote tissue regeneration and immunomodulation [7, 8]. Therefore, even transient persistence of MMSCs may be sufficient to initiate regenerative cascades that result in sustained functional improvement. Taken together, these findings suggest that the rapid clearance of MMSCs observed in normal immunodeficient animals reflects a context‐dependent mechanism of action rather than a limitation in therapeutic potential. In particular, the enhanced cell persistence observed in IC disease models supports the notion that inflammatory and regenerative microenvironments promote MMSC survival and therapeutic activity.

Notably, if the administered cells are neither detected in the gonads or reproductive organs nor associated with any abnormalities in these tissues, additional reproductive and developmental toxicity testing may not be warranted. Although the present study focused on safety evaluation, the favorable biodistribution, limited systemic exposure, and low immunogenicity of MMSCs support their potential for localized and sustained therapeutic effects. These safety characteristics, together with previously reported efficacy in IC models, provide a rationale for the clinical application of MMSCs.

Taken together, our findings offer a comprehensive pre‐clinical safety evaluation of the clinical‐grade MMSCs product, MR‐MC‐01. The product has been shown to be stable and does not promote any tumorigenic formation or migratory behavior when implanted in in vivo. Therefore, we concluded that our differentiation protocol yields a sufficiently pure population of terminally differentiated MSCs, effectively minimizing the risk of teratoma formation.

Advances in human development knowledge and improvements in hESC culture and differentiation protocols have enabled hESC derivatives to participate in the first clinical trials for several diseases, including IC (Table S11). Importantly, a Phase 1 clinical trial and a Phase 1/2a clinical trial of MMSCs (MR‐MC‐01) in patients with IC (ClinicalTrials.gov identifiers: NCT04610359 and KCT0006999, respectively) have been conducted. The available clinical data indicate that MMSCs are well tolerated, with no DLTs or serious treatment‐related adverse events observed [9, 45]. These clinical findings are consistent with the preclinical safety profile presented in this study, including the absence of tumorigenicity, restricted biodistribution to the injection site, and low immunogenicity of MMSCs. Together, these results support the translational relevance of our nonclinical safety evaluation and suggest that the favorable safety characteristics of MMSCs observed in preclinical models are maintained in human subjects. With stem cell‐based products advancing toward clinical application, these findings further support the potential of MMSCs as a safe and promising therapeutic option for IC.

## 5. Conclusions

In conclusion, our study provides robust and comprehensive nonclinical evidence supporting the clinical safety of hESC‐derived MMSCs intended for the treatment of IC. Extensive in vitro characterization confirmed the absence of residual pluripotent stem cell markers, while long‐term GLP‐compliant in vivo studies demonstrated no tumorigenicity, immunogenicity, or treatment‐related adverse effects following intra‐detrusor muscle administration at doses corresponding to the maximum feasible clinical exposure. Importantly, the lack of teratoma formation under clinically relevant conditions, together with clearly defined safety thresholds established through spiking studies, provides strong reassurance regarding the control of residual undifferentiated hESCs in the final product. Biodistribution analyses further support clinical applicability by demonstrating localized retention at the injection site and rapid systemic clearance without off‐target persistence.

Collectively, these findings establish a substantial safety margin and provide critical translational evidence supporting the advancement of hESC‐derived MMSCs into clinical evaluation. This work contributes to addressing longstanding safety concerns surrounding pluripotent stem cell–derived therapies and supports their development as a viable and scalable cell‐based therapeutic strategy for patients with IC.

## Funding

This present work was financially supported by the Korean Fund for Regenerative Medicine (KFRM) grant funded by the Korea government (the Ministry of Science and ICT, the Ministry of Health & Welfare) (Grant 21D0804L1), by Jeollanam‐do and Jeonnam Bio Foundation (JBF), Republic of Korea, and by the Regional Innovation System & Education (RISE) program through the Jeju RISE center, funded by the Ministry of Education (MOE) and the Jeju Special Self‐Governing Province, Republic of Korea (Grant 2026‐RISE‐17‐001).

## Conflicts of Interest

The authors declare no conflicts of interest.

## Supporting Information

Additional supporting information can be found online in the Supporting Information section.

## Supporting information


**Supporting Information** Table S1. Testing of GMP grade hESC‐MCB. Table S2. Testing of GMP grade MMSCs. Table S3. Group allocations in general toxicity study. Table S4. Group allocations in the tumorigenicity study. Table S5. Group allocations in the hESC spiking study. Table S6. Group allocations in the biodistribution study. Figure S1. Genomic stability of MMSCs assessed by CytoScan HD and InfiniumOmni5‐4 arrays. Genome‐wide copy number variation (CNV) and single nucleotide polymorphism (SNP) profiles demonstrate the genetic stability of the working cell bank (WCB) and final MMSC products (three lots). (A–E) CytoScan HD array results for the final product (Passage 7), showing the karyoview (A), B allele frequency and weighted Log2 ratio (B, C), allelic difference (D), and smooth signal (E). (CNV filtering criteria: segment size ≥25 kb, marker count ≥10, excluding DGV variants). (F–I) Individual chromosomal plots from the InfiniumOmni5‐4 array for the WCB (F) and final products (G–I). Consistent Log R ratio and B allele frequency profiles across all samples reveal no significant copy number alterations, confirming the robust maintenance of a normal diploid karyotype. (J) Plots of aberration frequency by sample showing high copy gain, copy gain, copy loss, and homozygous loss events. Abbreviations: MSC‐W‐0006, working cell bank (WCB); MSP‐0005, MSP‐0006, MSP‐0007, and MSP‐0009, final product MMSCs. Table S7. Clinical biochemistry test in the general toxicity test. Table S8. Primer and probe sequence used for qRT‐PCR in the biodistribution study. Table S9. MMSC cell numbers in urinary bladders calculated by qRT‐PCR method. Table S10. Frequency of teratoma formation in mice injected with MMSCs or MMSCs spiked with increasing levels of undifferentiated hESCs. Table S11. Summary of cell therapies based on hESC‐derived mesenchymal stem cells, found in the text.

## Data Availability

The data that support the findings of this study are available from the corresponding author upon reasonable request.
