# Supplementary material for: Comprehensive Preclinical Safety Evaluation of Clinical‐Grade hESC‐Derived Multipotent Mesenchymal Stem Cells for Interstitial Cystitis
Source: Stem Cells Int. 2026 Aug 18;2026:5539154. doi: 10.1155/sci/5539154 (PMC13484282; doi:10.1155/sci/5539154)
Supplement: Supplementary file 1 [file SCI-2026-5539154-s001.zip › Supporting Information 1. Table S1.docx]

Table S1. Testing of GMP grade hESC-MCB

| **Tests** | **Results** |
| --- | --- |
| *In vitro* assay for adventitious viral contaminants | Negative |
| *In vivo* assay for adventitious virus: | |
| Embryonated hens’ eggs | Negative |
| Suckling mice | Negative |
| Human T-cell lymphotrophic virus 1 DNA by qRT-PCR | Negative |
| Human T-cell lymphotrophic virus 2 DNA by qRT-PCR | Negative |
| Hepatitis A Virus (HAV) RNA by qRT-PCR | Negative |
| Hepatitis B Virus (HBV) DNA by qRT-PCR | Negative |
| Hepatitis C Virus (HCV) RNA by qRT-PCR | Negative |
| Human immuno-deficiency virus type-1 (HIV-1) RNA by qRT-PCR | Negative |
| Human immuno-deficiency virus type-2 (HIV-2) RNA by qRT-PCR | Negative |
| Polymerase Chain Reaction (PCR)-Based Reverse Transcription Assay (PERT) for Retrovirus Detection | Negative |
| Mycoplasma (28 day) | Negative |
| Sterility (14 day) | Negative |
