# Supplementary material for: Comprehensive Preclinical Safety Evaluation of Clinical‐Grade hESC‐Derived Multipotent Mesenchymal Stem Cells for Interstitial Cystitis
Source: Stem Cells Int. 2026 Aug 18;2026:5539154. doi: 10.1155/sci/5539154 (PMC13484282; doi:10.1155/sci/5539154)
Supplement: Supplementary file 1 [file SCI-2026-5539154-s001.zip › Supporting Information 10. Table S9.docx]

Table S9. MMSC cell numbers in urinary bladders calculated by qRT-PCR method

| **Male** | **12 hr** | **Day 1** | **Day 3** | **Day 10** | **Week 4** | **Week 8** | **Week 12** | **Week 26** | **Week 52** |
| --- | --- | --- | --- | --- | --- | --- | --- | --- | --- |
| #1 | 2.851 | 0.173 | 0.422 | LLOQ | LLOQ | LLOQ | LLOQ | LLOQ | LLOQ |
| #2 | 1.184 | 0.198 | 0.019 | LLOQ | LLOQ | LLOQ | LLOQ | LLOQ | LLOQ |
| #3 | 1.507 | 0.624 | 0.006 | LLOQ | LLOQ | LLOQ | LLOQ | LLOQ | LLOQ |
| Ave | 1.847 | 0.332 | 0.149 | LLOQ | LLOQ | LLOQ | LLOQ | LLOQ | LLOQ |
| S.E | 0.510 | 0.146 | 0.137 | LLOQ | LLOQ | LLOQ | LLOQ | LLOQ | LLOQ |
| **Female** | **12 hr** | **Day 1** | **Day 3** | **Day 10** | **Week 4** | **Week 8** | **Week 12** | **Week 26** | **Week 52** |
| #1 | 2.473 | 0.343 | 0.199 | LLOQ | LLOQ | LLOQ | LLOQ | LLOQ | LLOQ |
| #2 | 1.585 | 0.436 | 0.001 | LLOQ | LLOQ | LLOQ | LLOQ | LLOQ | LLOQ |
| #3 | 0.891 | 0.180 | 0.026 | LLOQ | LLOQ | LLOQ | LLOQ | LLOQ | LLOQ |
| Ave | 1.650 | 0.319 | 0.075 | LLOQ | LLOQ | LLOQ | LLOQ | LLOQ | LLOQ |
| S.E | 0.458 | 0.075 | 0.062 | LLOQ | LLOQ | LLOQ | LLOQ | LLOQ | LLOQ |

( **×** 10^5^ cells/urinary bladders)

Data are presented as the mean ± SE

LLOQ: lower limit of quantification
