# Supplementary material for: Comprehensive Preclinical Safety Evaluation of Clinical‐Grade hESC‐Derived Multipotent Mesenchymal Stem Cells for Interstitial Cystitis
Source: Stem Cells Int. 2026 Aug 18;2026:5539154. doi: 10.1155/sci/5539154 (PMC13484282; doi:10.1155/sci/5539154)
Supplement: Supplementary file 1 [file SCI-2026-5539154-s001.zip › Supporting Information 11. Table S10.docx]

Table S10. Frequency of teratoma formation in mice injected with MMSCs or MMSCs spiked with increasing levels of undifferentiated hESCs

| **Male** | **Groups** | | | | | | |
| --- | --- | --- | --- | --- | --- | --- | --- |
|  | **G1** | **G2** | **G3** | **G4** | **G5** | **G6** | **G7^a^** |
| Number of animals | 15 | 15 | 15 | 15 | 15 | 15 | 15 |
| Histiocytic sarcoma | - | - | - | - | - | - | - |
| Lymphoma | 2 | 2 | 3 | 4 | 2 | 3 | 4 |
| Leukemia | - | - | - | - | - | 1 | - |
| Hemangioma | - | - | 1 | - | - | - | - |
| Chondroma | 1 | - | - | - | - | - | - |
| Adenoma/adenocarcinoma | 2 | 1 | - | - | 2 | - | - |
| Polyp | 1 | - | 1 | 1 | 1 | - | - |
| Teratoma : Injection site  (s.c. or intra-detrusor muscle) | - | 10 | 8 | 9 | 5 | - | - |
| **Female** | **Groups** | | | | | | |
|  | **G1** | **G2** | **G3** | **G4** | **G5** | **G6** | **G7^a^** |
| Number of animals | 15 | 15 | 15 | 15 | 15 | 15 | 15 |
| Histiocytic sarcoma | 1 | - | 1 | - | - | - | - |
| Lymphoma | 9 | 5 | 9 | 3 | 6 | 6 | 2 |
| Leukemia | - | 2 | 1 | 2 | - | 3 | 1 |
| Hemangioma | - | - | - | - | - | - | - |
| Chondroma | - | - | - | - | - | - | - |
| Adenoma/adenocarcinoma | - | - | - | - | - | 1 | 1 |
| Polyp | - | 1 | - | - | - | - | - |
| Teratoma : Injection site  (s.c. or intra-detrusor muscle) | - | 6 | 7 | 6 | 6 | - | - |

G1: Vehicle (s.c. + intra-detrusor muscle)

G2: 100% hESCs (s.c.)

G3: 1% hESCs + 99% MMSCs (s.c.)

G4: 2% hESCs + 98% MMSCs (s.c.)

G5: 2% hESCs + 98% MMSCs (intra-detrusor muscle)

G6: 100% MMSCs (intra-detrusor muscle)

G7: 2% hESCs + 98% MMSCs (intra-detrusor muscle)

^a^Cells were injected without Matrigel.
