# Supplementary material for: Comprehensive Preclinical Safety Evaluation of Clinical‐Grade hESC‐Derived Multipotent Mesenchymal Stem Cells for Interstitial Cystitis
Source: Stem Cells Int. 2026 Aug 18;2026:5539154. doi: 10.1155/sci/5539154 (PMC13484282; doi:10.1155/sci/5539154)
Supplement: Supplementary file 1 [file SCI-2026-5539154-s001.zip › Supporting Information 12. Table S11.docx]

Table S11. Summary of cell therapies based on hESC-derived mesenchymal stem cells, found in the text

| **Biological** | **Conditions** | **Clinical phase** | **Sponsor** | **Start date** | **Study status** | **Reference**  **number** |
| --- | --- | --- | --- | --- | --- | --- |
| hESC derived MSC like cell | Meniscus injury | Phase 1 | Chinese Academy of Sciences | 2019-01-04 | Unknown | NCT03839238 |
| hESC derived MSC like cell | Primary ovarian insufficiency | Phase 1 | Chinese Academy of Sciences | 2019-04-03 | Unknown | NCT03877471 |
| hESC derived mesenchymal cells | Intrauterine adhesion | Phase 1 | Chinese Academy of Sciences | 2020-01-01 | Unknown | NCT04232592 |
| CAStem | COVID-19 acute respiratory distress syndrome virus; pneumonia acute lung injury | Phase 1,  Phase 2 | Chinese Academy of Sciences | 2020-01-27 | Unknown | NCT04331613 |
| IMS001-01 | Multiple sclerosis | Phase 1 | ImStem Biotechnology | 2021-08-31 | Recruiting | NCT04956744 |
| MR-MC-01  (MMSC) | Interstitial cystitis | Phase 1 | Asan Medical Center | 2020-10-20 | Completed | NCT04610359 |
| MR-MC-01  (MMSC) | Interstitial cystitis | Phase 1/2a | Mirae Cell Bio | 2021-11-22 | Completed | KCT0006999 |
| MR-MC-01  (MMSC) | Underactive bladder | Phase 1/2a | Mirae Cell Bio | 2023-09-25 | Recruiting | KCT0009028 |
