# Supplementary material for: Comprehensive Preclinical Safety Evaluation of Clinical‐Grade hESC‐Derived Multipotent Mesenchymal Stem Cells for Interstitial Cystitis
Source: Stem Cells Int. 2026 Aug 18;2026:5539154. doi: 10.1155/sci/5539154 (PMC13484282; doi:10.1155/sci/5539154)
Supplement: Supplementary file 1 [file SCI-2026-5539154-s001.zip › Supporting Information 2. Table S2.docx]

Table S2. Testing of GMP grade MMSCs

| **Tests** | **Results** |
| --- | --- |
| *In vitro* assay for adventitious viral contaminants | Negative |
| Mycoplasma (28 day) | Negative |
| Sterility (14 day) | Negative |
| Endotoxin test (kinetic assay) | < 0.5 EU/mL |
