# Supplementary material for: Comprehensive Preclinical Safety Evaluation of Clinical‐Grade hESC‐Derived Multipotent Mesenchymal Stem Cells for Interstitial Cystitis
Source: Stem Cells Int. 2026 Aug 18;2026:5539154. doi: 10.1155/sci/5539154 (PMC13484282; doi:10.1155/sci/5539154)
Supplement: Supplementary file 1 [file SCI-2026-5539154-s001.zip › Supporting Information 3. Table S3.docx]

Table S3. Group allocations in general toxicity study

| **Groups** | **Mouse No.**  **and sex** | **Injection site** | **Total injection volume**  **(mL/head)** | **Total dose per mouse**  **(cells/head)** |
| --- | --- | --- | --- | --- |
| G1 | M 10 / F 10 | Intra-detrusor muscle | 0.1 | 0 |
| G2 | M 10 / F 10 | Intra-detrusor muscle | 0.1 | 8 × 10^4^ |
| G3 | M 10 / F 10 | Intra-detrusor muscle | 0.1 | 4 × 10^5^ |
| G4 | M 10 / F 10 | Intra-detrusor muscle | 0.1 | 2 × 10^6^ |

*Observation period: 13 weeks
