# Supplementary material for: Comprehensive Preclinical Safety Evaluation of Clinical‐Grade hESC‐Derived Multipotent Mesenchymal Stem Cells for Interstitial Cystitis
Source: Stem Cells Int. 2026 Aug 18;2026:5539154. doi: 10.1155/sci/5539154 (PMC13484282; doi:10.1155/sci/5539154)
Supplement: Supplementary file 1 [file SCI-2026-5539154-s001.zip › Supporting Information 4. Table S4.docx]

Table S4. Group allocations in the tumorigenicity study

| **Groups** | **Mouse No. and sex** | **Total injection volume**  **(mL/head)** | **Injection sites** | **Total dose per mouse**  **(cells/head)** | | |
| --- | --- | --- | --- | --- | --- | --- |
|  |  |  |  | **MMSCs** | **HeLa cells** | **Total** |
| G1 | M 10 / F 10 | 0.2  + 0.1  (2 sites) | Subcutaneous  + intra-detrusor muscle | 0 | 0 | 0 |
| G2 | M 10 / F 10 | 0.2 | Subcutaneous | 0 | 1 × 10^7^ | 1 × 10^7^ |
| G3 | M 10 / F 10 | 0.2 | Subcutaneous | 1 × 10^7^ | 0 | 1 × 10^7^ |
| G4 | M 10 / F 10 | 0.1 | Intra-detrusor muscle | 2 × 10^6^ | 0 | 2 × 10^6^ |

*Observation period: 52 weeks
