# Supplementary material for: Comprehensive Preclinical Safety Evaluation of Clinical‐Grade hESC‐Derived Multipotent Mesenchymal Stem Cells for Interstitial Cystitis
Source: Stem Cells Int. 2026 Aug 18;2026:5539154. doi: 10.1155/sci/5539154 (PMC13484282; doi:10.1155/sci/5539154)
Supplement: Supplementary file 1 [file SCI-2026-5539154-s001.zip › Supporting Information 5. Table S5.docx]

Table S5. Group allocations in the hESC spiking study

| **Groups** | **Mouse No. and sex** | **Total injection volume**  **(mL/head)** | **Injection sites** | **Spiking group** | **Total dose per mouse**  **(cells/head)** | | |
| --- | --- | --- | --- | --- | --- | --- | --- |
|  |  |  |  |  | **MMSCs** | **hESCs** | **Total** |
| G1 | M 15 / F 15 | 0.2  + 0.1  (2 sites) | Subcutaneous  + intra-detrusor muscle | Vehicle | 0 | 0 | 0 |
| G2 | M 15 / F 15 | 0.2 | Subcutaneous | 100% hESCs | 0 | 2 × 10^6^ | 2 × 10^6^ |
| G3 | M 15 / F 15 | 0.2 | Subcutaneous | 1% hESCs + 99% MMSCs | 1.98 × 10^6^ | 2 × 10^4^ | 2 × 10^6^ |
| G4 | M 15 / F 15 | 0.2 | Subcutaneous | 2% hESCs + 98% MMSCs | 1.96 × 10^6^ | 4 × 10^4^ | 2 × 10^6^ |
| G5 | M 15 / F 15 | 0.1  (2 sites) | Intra-detrusor muscle | 2% hESCs + 98% MMSCs | 1.96 × 10^6^ | 4 × 10^4^ | 2 × 10^6^ |
| G6 | M 15 / F 15 | 0.1  (2 sites) | Intra-detrusor muscle | 100% MMSCs | 2 × 10^6^ | 0 | 2 × 10^6^ |
| G7^a^ | M 15 / F 15 | 0.1  (2 sites) | Intra-detrusor muscle | 2% hESCs + 98% MMSCs | 1.96 × 10^6^ | 4 × 10^4^ | 2 × 10^6^ |

*Observation period: 52 weeks

^a^Cells were injected without Matrigel.
