# Supplementary material for: Comprehensive Preclinical Safety Evaluation of Clinical‐Grade hESC‐Derived Multipotent Mesenchymal Stem Cells for Interstitial Cystitis
Source: Stem Cells Int. 2026 Aug 18;2026:5539154. doi: 10.1155/sci/5539154 (PMC13484282; doi:10.1155/sci/5539154)
Supplement: Supplementary file 1 [file SCI-2026-5539154-s001.zip › Supporting Information 6. Table S6.docx]

Table S6. Group allocations in the biodistribution study

| **Groups** | | **mouse No.**  **and sex** | **Total injection volume**  **(mL/head)** | **Total dose per mouse**  **(cells/head)** |
| --- | --- | --- | --- | --- |
| G1 | Vehicle control | M 3 / F 3 | 0.1 | 2 × 10^6^ |
| G2 | 12 hrs after administration | M 3 / F 3 | 0.1 | 2 × 10^6^ |
| G3 | Day 1 after administration | M 3 / F 3 | 0.1 | 2 × 10^6^ |
| G4 | Day 3 after administration | M 3 / F 3 | 0.1 | 2 × 10^6^ |
| G5 | Day 10 after administration | M 3 / F 3 | 0.1 | 2 × 10^6^ |
| G6 | Week 4 after administration | M 3 / F 3 | 0.1 | 2 × 10^6^ |
| G7 | Week 8 after administration | M 3 / F 3 | 0.1 | 2 × 10^6^ |
| G8 | Week 12 after administration | M 3 / F 3 | 0.1 | 2 × 10^6^ |
| G9 | Week 26 after administration | M 3 / F 3 | 0.1 | 2 × 10^6^ |
| G10 | Week 52 after administration | M 3 / F 3 | 0.1 | 2 × 10^6^ |
