# Supplementary material for: Comprehensive Preclinical Safety Evaluation of Clinical‐Grade hESC‐Derived Multipotent Mesenchymal Stem Cells for Interstitial Cystitis
Source: Stem Cells Int. 2026 Aug 18;2026:5539154. doi: 10.1155/sci/5539154 (PMC13484282; doi:10.1155/sci/5539154)
Supplement: Supplementary file 1 [file SCI-2026-5539154-s001.zip › Supporting Information 7. Figure S1.pdf]

A

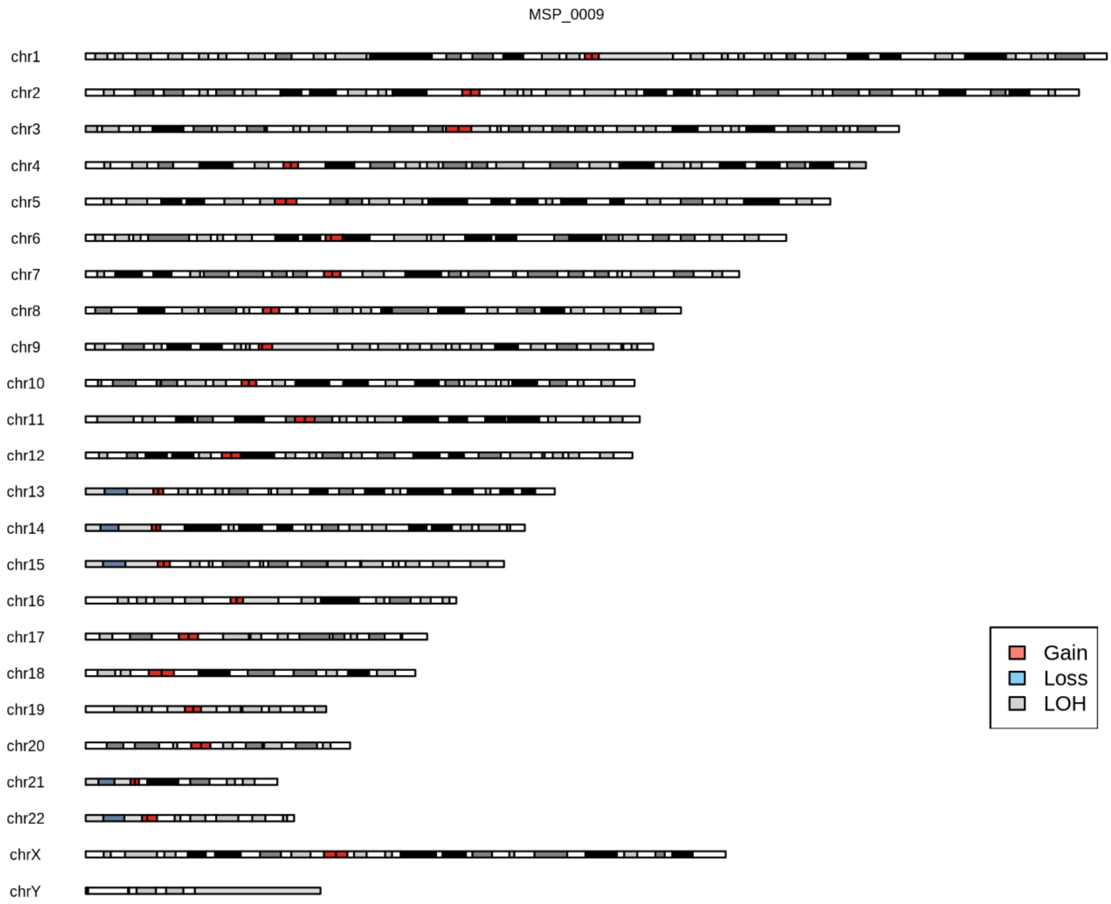
MSP_0009
chr1
chr2
chr3
chr4
chr5
chr6
chr7
chr8
chr9
chr10
chr11
chr12
chr13
chr14
chr15
chr16
chr17
chr18
chr19
chr20
chr21
chr22
chrX
chrY
Gain
Loss
LOH

B

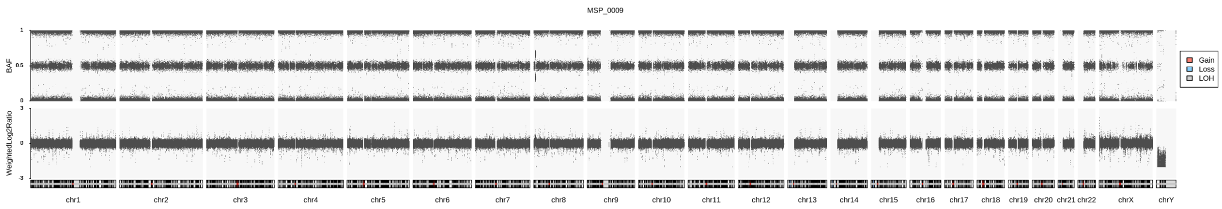
MSP_0009
BAF
WeightedLog2Ratio
Gain
Loss
LOH
chr1
chr2
chr3
chr4
chr5
chr6
chr7
chr8
chr9
chr10
chr11
chr12
chr13
chr14
chr15
chr16
chr17
chr18
chr19
chr20
chr21
chr22
chrX
chrY

C

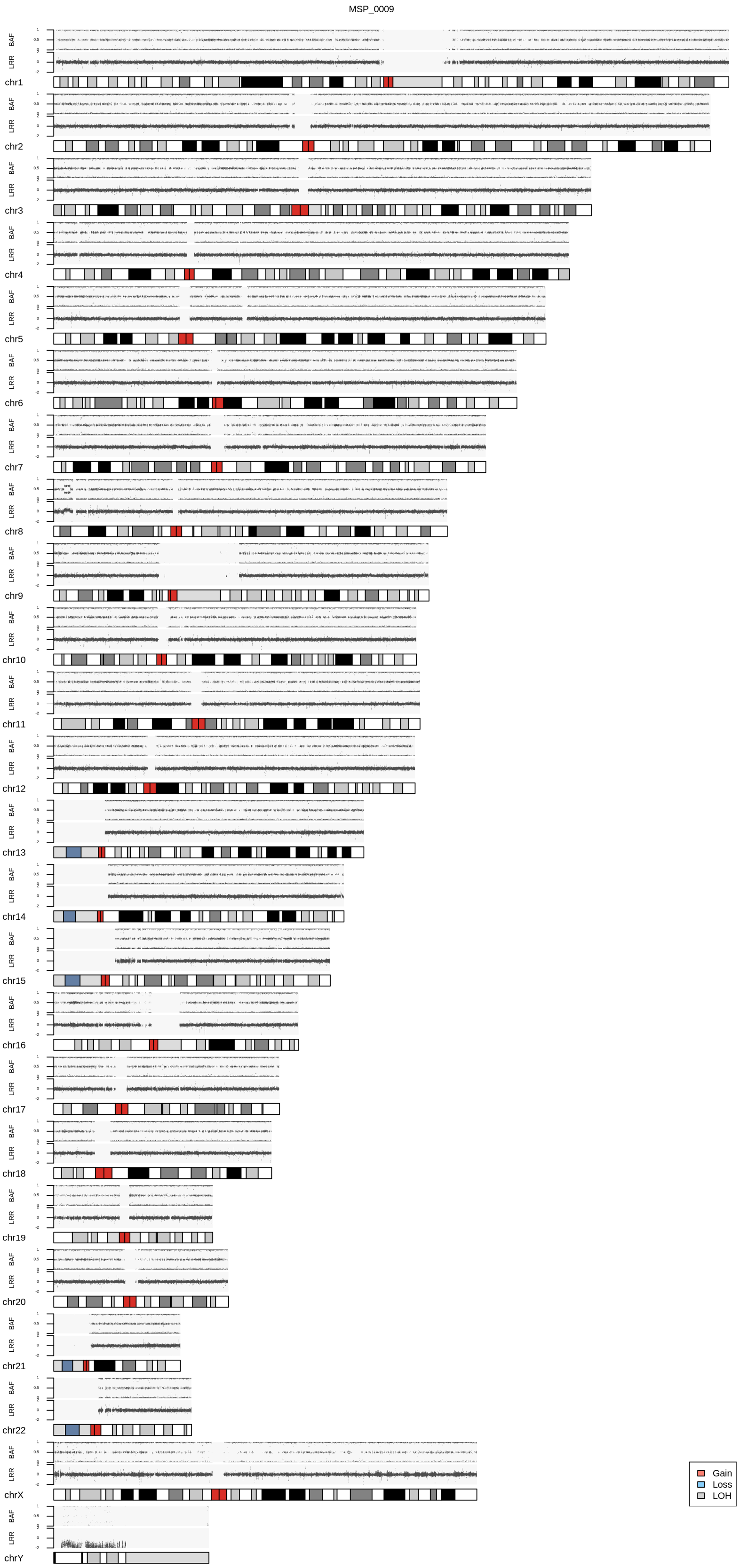

MSP_0009
chr1
chr2
chr3
chr4
chr5
chr6
chr7
chr8
chr9
chr10
chr11
chr12
chr13
chr14
chr15
chr16
chr17
chr18
chr19
chr20
chr21
chr22
chrX
chrY
BAF
LRR
Gain
Loss
LOH

D

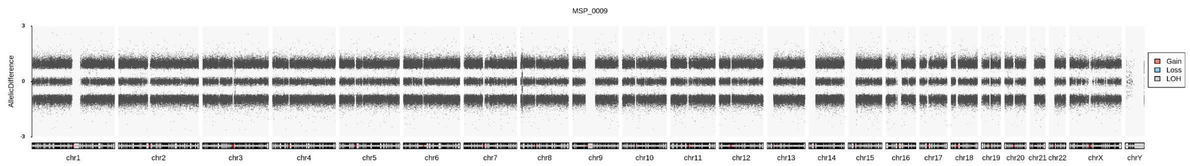
MSP_0009
AllelicDifference
3
0
-3
chr1 chr2 chr3 chr4 chr5 chr6 chr7 chr8 chr9 chr10 chr11 chr12 chr13 chr14 chr15 chr16 chr17 chr18 chr19 chr20 chr21 chr22 chrX chrY
Gain
Loss
LOH

E

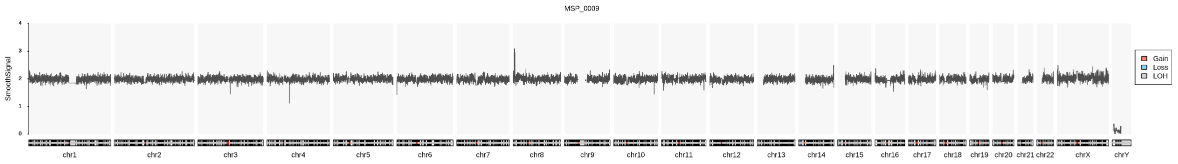
MSP_0009
SmoothSignal
4
3
2
1
0
chr1 chr2 chr3 chr4 chr5 chr6 chr7 chr8 chr9 chr10 chr11 chr12 chr13 chr14 chr15 chr16 chr17 chr18 chr19 chr20 chr21 chr22 chrX chrY
Gain
Loss
LOH

F

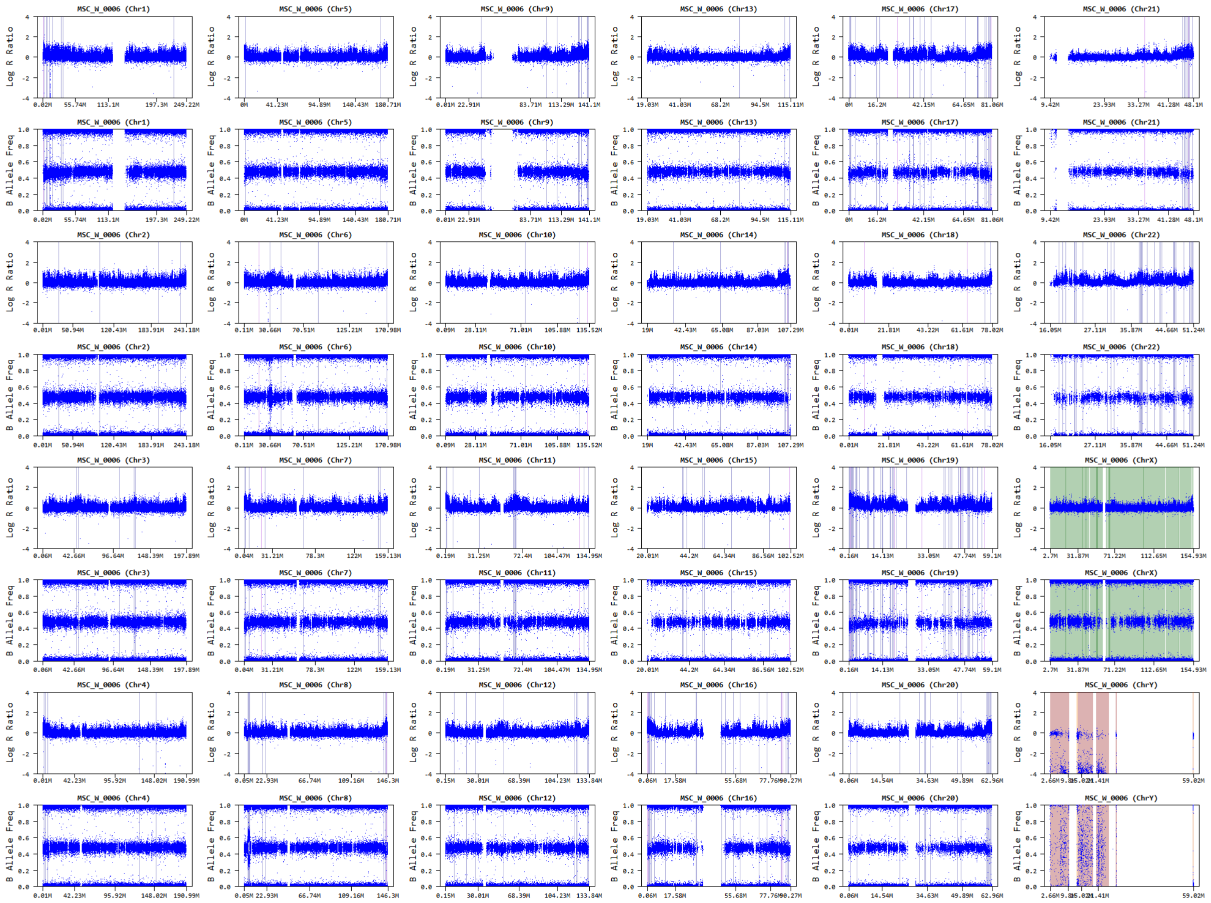
MSC_W_0006 (Chr1)
MSC_W_0006 (Chr5)
MSC_W_0006 (Chr9)
MSC_W_0006 (Chr13)
MSC_W_0006 (Chr17)
MSC_W_0006 (Chr21)
MSC_W_0006 (Chr2)
MSC_W_0006 (Chr6)
MSC_W_0006 (Chr10)
MSC_W_0006 (Chr14)
MSC_W_0006 (Chr18)
MSC_W_0006 (Chr22)
MSC_W_0006 (Chr3)
MSC_W_0006 (Chr7)
MSC_W_0006 (Chr11)
MSC_W_0006 (Chr15)
MSC_W_0006 (Chr19)
MSC_W_0006 (ChrX)
MSC_W_0006 (Chr4)
MSC_W_0006 (Chr8)
MSC_W_0006 (Chr12)
MSC_W_0006 (Chr16)
MSC_W_0006 (Chr20)
MSC_W_0006 (ChrY)
Log R Ratio
B Allele Freq

G

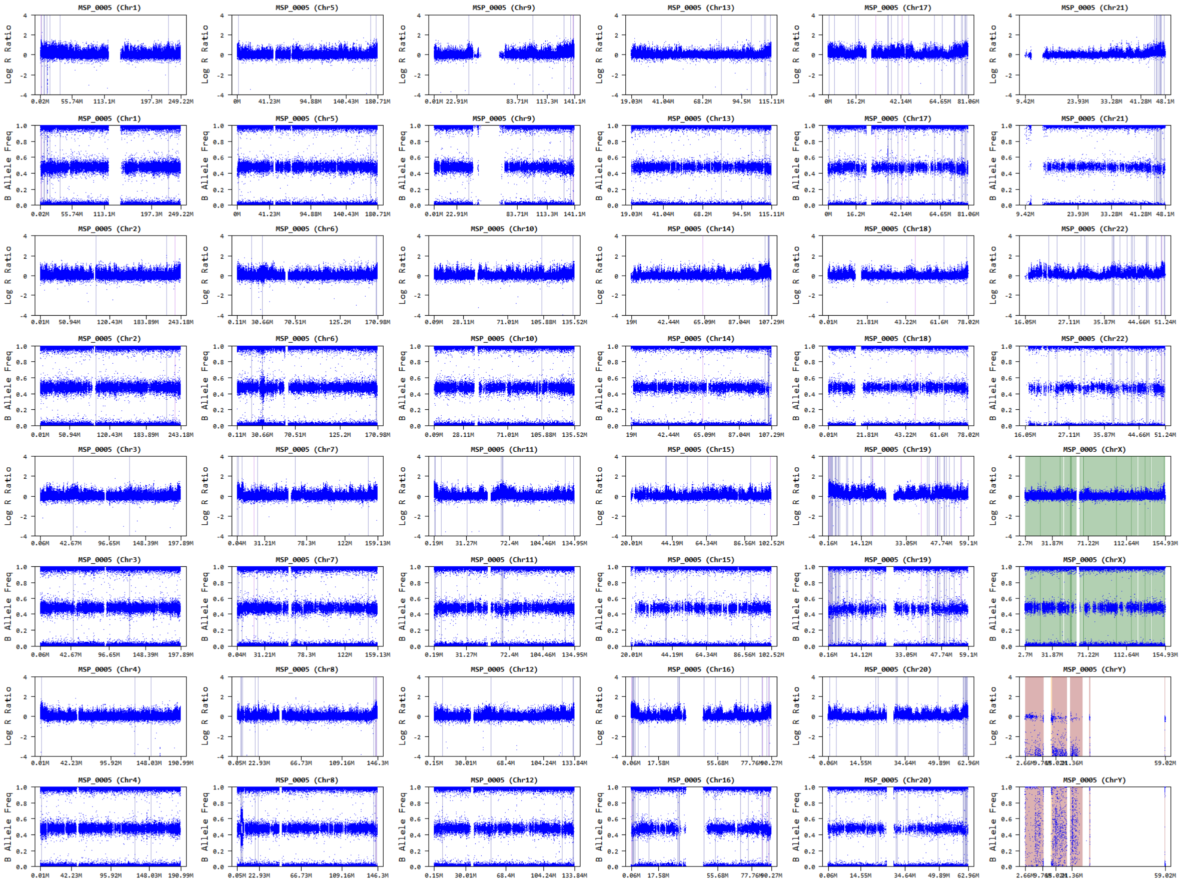
MSP_0005 (Chr1)
MSP_0005 (Chr5)
MSP_0005 (Chr9)
MSP_0005 (Chr13)
MSP_0005 (Chr17)
MSP_0005 (Chr21)
MSP_0005 (Chr2)
MSP_0005 (Chr6)
MSP_0005 (Chr10)
MSP_0005 (Chr14)
MSP_0005 (Chr18)
MSP_0005 (Chr22)
MSP_0005 (Chr3)
MSP_0005 (Chr7)
MSP_0005 (Chr11)
MSP_0005 (Chr15)
MSP_0005 (Chr19)
MSP_0005 (ChrX)
MSP_0005 (Chr4)
MSP_0005 (Chr8)
MSP_0005 (Chr12)
MSP_0005 (Chr16)
MSP_0005 (Chr20)
MSP_0005 (ChrY)
Log R Ratio
B Allele Freq

H

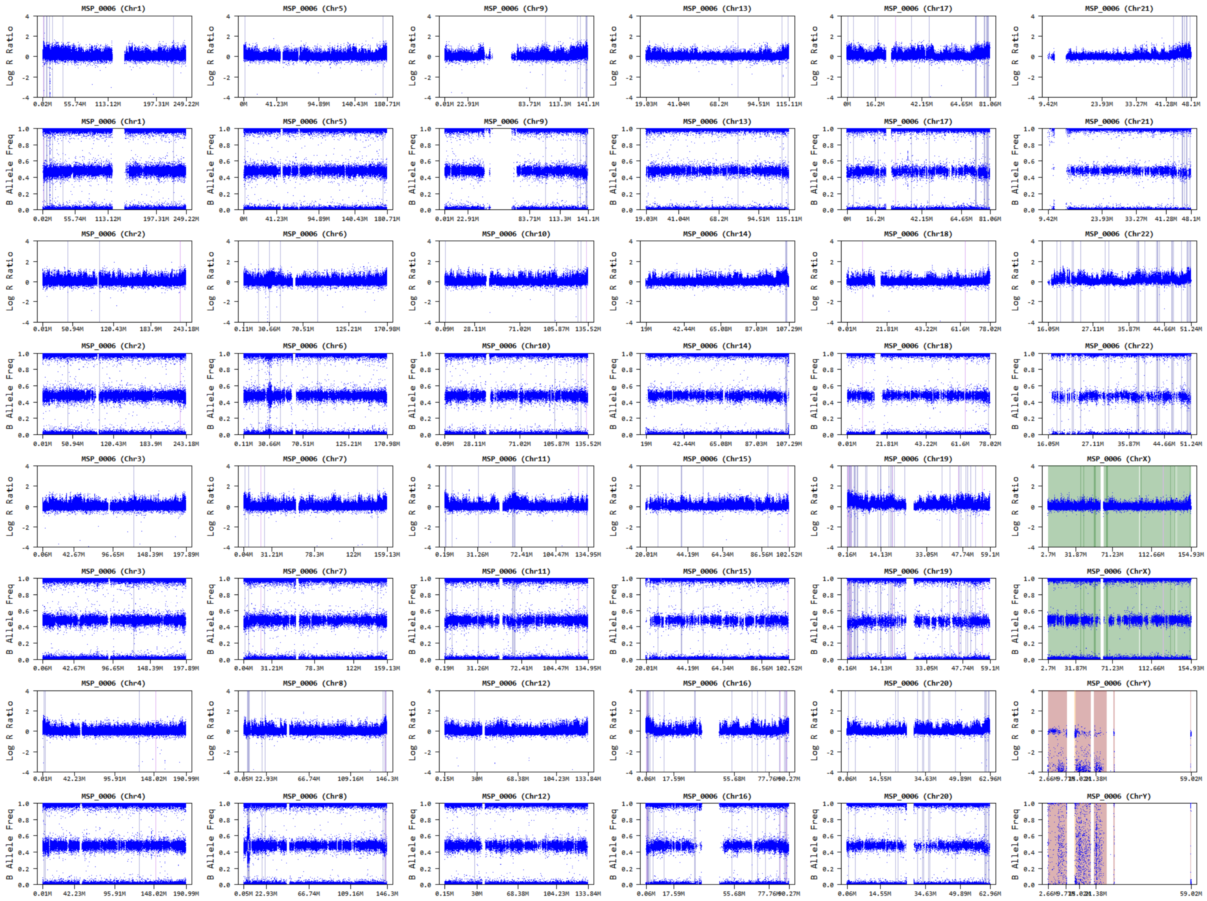

MSP_0006 (Chr1)
MSP_0006 (Chr5)
MSP_0006 (Chr9)
MSP_0006 (Chr13)
MSP_0006 (Chr17)
MSP_0006 (Chr21)
MSP_0006 (Chr2)
MSP_0006 (Chr6)
MSP_0006 (Chr10)
MSP_0006 (Chr14)
MSP_0006 (Chr18)
MSP_0006 (Chr22)
MSP_0006 (Chr3)
MSP_0006 (Chr7)
MSP_0006 (Chr11)
MSP_0006 (Chr15)
MSP_0006 (Chr19)
MSP_0006 (ChrX)
MSP_0006 (Chr4)
MSP_0006 (Chr8)
MSP_0006 (Chr12)
MSP_0006 (Chr16)
MSP_0006 (Chr20)
MSP_0006 (ChrY)
Log R Ratio
B Allele Freq

I

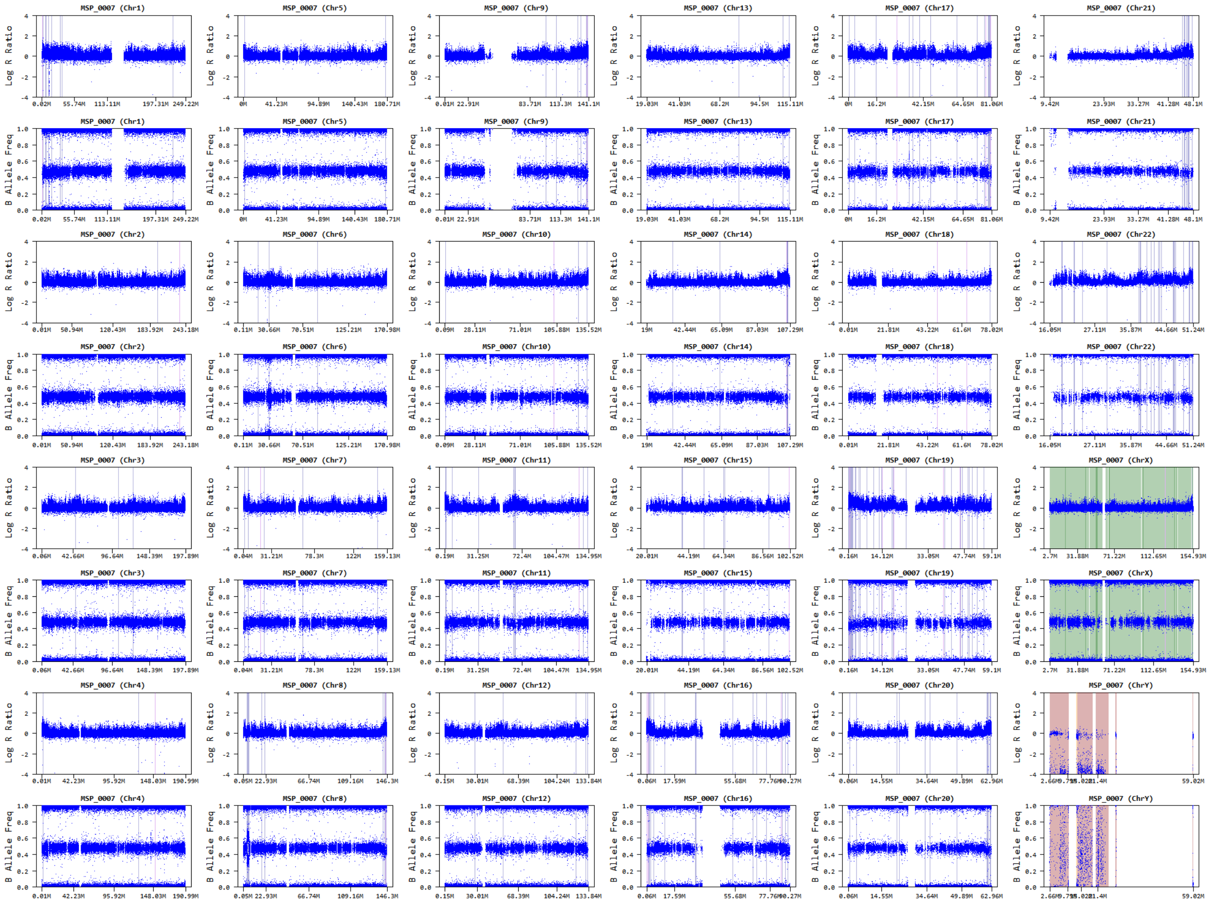

J

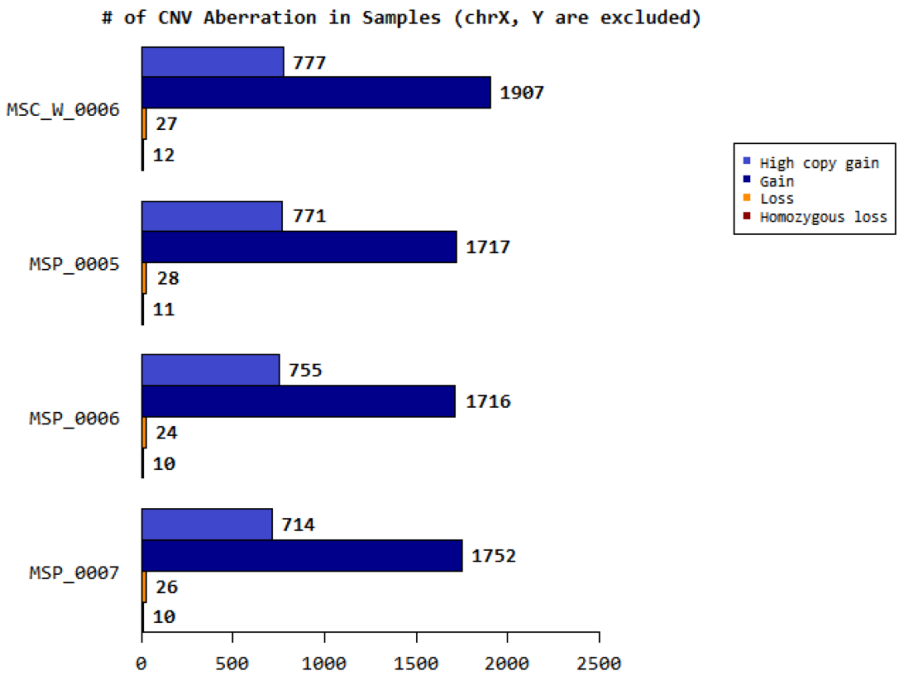

Figure S1. Genomic stability of MMSCs assessed by CytoScan HD and InfiniumOmni5-4 arrays. Genome-wide copy number variation (CNV) and single nucleotide polymorphism (SNP) profiles demonstrate the genetic stability of the working cell bank (WCB) and final MMSC products (three lots). (A–E) CytoScan HD array results for the final product (Passage 7), showing the karyoview (A), B allele frequency and weighted Log2 ratio (B, C), allelic difference (D), and smooth signal (E). (CNV filtering criteria: segment size ≥25 kb, marker count ≥10, excluding DGV variants). (F–I) Individual chromosomal plots from the InfiniumOmni5-4 array for the WCB (F) and final products (G–I). Consistent Log R ratio and B allele frequency profiles across all samples reveal no significant copy number alterations, confirming the robust maintenance of a normal diploid karyotype. (J) Plots of aberration frequency by sample showing high copy gain, copy gain, copy loss, and homozygous loss events. Abbreviations: MSC-W-0006, working cell bank (WCB); MSP-0005, MSP-0006, MSP-0007, and MSP-0009, final product MMSCs.
