# Supplementary material for: Comprehensive Preclinical Safety Evaluation of Clinical‐Grade hESC‐Derived Multipotent Mesenchymal Stem Cells for Interstitial Cystitis
Source: Stem Cells Int. 2026 Aug 18;2026:5539154. doi: 10.1155/sci/5539154 (PMC13484282; doi:10.1155/sci/5539154)
Supplement: Supplementary file 1 [file SCI-2026-5539154-s001.zip › Supporting Information 8. Table S7.docx]

Table S7. Clinical biochemistry test in the general toxicity test

| **Male** | | | | | |
| --- | --- | --- | --- | --- | --- |
| **Tests** | **Units** | **Groups (cells/head)** | | | |
|  |  | **G1 (0)** | **G2 (8 × 10^4^)** | **G3 (4 × 10^5^)** | **G4 (2 × 10^6^)** |
| AST | U/L | 93.1 ± 26.6 | 79.7 ± 32.3 | 76.2 ± 11.7 | 78.1 ± 26.2 |
| ALT | U/L | 52.3 ± 30.1 | 40.7 ± 20.9 | 39.3 ± 13.7 | 34.6 ± 8.7 |
| ALP | U/L | 76.7 ± 17.5 | 76.9 ± 13.1 | 74.8 ± 6.0 | 78.5 ± 11.2 |
| CPK | U/L | 145.7 ± 56.0 | 126.3 ± 90.1 | 99.6 ± 49.6 | 101.7 ± 63.9 |
| TBIL | mg/dL | 0.124 ± 0.029 | 0.143 ± 0.031 | 0.124 ± 0.037 | 0.127 ± 0.023 |
| GLU | mg/dL | 192.4 ± 23.2 | 236.0 ± 49.5 | 224.4 ± 42.4 | 216.9 ± 25.9 |
| TCHO | mg/dL | 112.1 ± 16.4 | 116.9 ± 22.5 | 117.5 ± 10.3 | 119.9 ± 11.6 |
| TG | mg/dL | 64.1 ± 10.7 | 79.8 ± 26.9 | 72.2 ± 16.9 | 76.2 ± 10.2 |
| TP | g/dL | 5.24 ± 0.25 | 5.40 ± 0.76 | 5.21 ± 0.26 | 5.24 ± 0.25 |
| ALB | g/dL | 2.18 ± 0.27 | 2.23 ± 0.36 | 2.38 ± 0.13 | 2.39 ± 0.08 |
| A/G ratio | - | 1.02 ± 0.82 | 0.72 ± 0.14 | 0.85 ± 0.11 | 0.84 ± 0.05 |
| BUN | mg/dL | 20.4 ± 2.4 | 24.2 ± 4.8 | 21.0 ± 2.9 | 21.9 ± 5.1 |
| CRE | mg/dL | 0.29 ± 0.04 | 0.27 ± 0.06 | 0.28 ± 0.02 | 0.28 ± 0.03 |
| IP | mg/dL | 7.23 ± 1.00 | 6.64 ± 1.53 | 7.24 ± 1.54 | 7.99 ± 1.93 |
| Ca^2+^ | mg/dL | 8.66 ± 0.57 | 8.6 ± 1.33 | 8.96 ± 0.44 | 9.18 ± 0.38 |
| Na^+^ | mmol/L | 145.7 ± 9.2 | 147.6 ± 18.2 | 150.3 ± 4.0 | 152.2 ± 1.7 |
| K^+^ | mmol/L | 5.08 ± 0.84 | 4.81 ± 0.51 | 5.32 ± 1.09 | 6.05 ± 2.07 |
| Cl^-^ | mmol/L | 115.4 ± 4.7 | 116.4 ± 14.4 | 117.2 ± 3.2 | 118.3 ± 2.4 |
| Number of animals | | 9 | 9 | 10 | 10 |
| **Female** | | | | | |
| **Tests** | **Units** | **Groups (cells/head)** | | | |
|  |  | **G1 (0)** | **G2 (8 × 10^4^)** | **G3 (4 × 10^5^)** | **G4 (2 × 10^6^)** |
| AST | U/L | 92.8 ± 30.8 | 78.2 ± 10.3 | 107.7 ± 64.4 | 79.7 ± 11.3 |
| ALT | U/L | 31.9 ± 10.4 | 26.6 ± 6.1 | 58.2 ± 64.1 | 29.8 ± 5.5 |
| ALP | U/L | 63.3 ± 14.0 | 67.9 ± 17.1 | 70.9 ± 9.3 | 78.3 ± 9.3 |
| CPK | U/L | 108.6 ± 55.4 | 97.5 ± 37.3 | 84.9 ± 26.8 | 106.5 ± 37.2 |
| TBIL | mg/dL | 0.085 ± 0.035 | 0.095 ± 0.043 | 0.096 ± 0.043 | 0.073 ± 0.056 |
| GLU | mg/dL | 205.4 ± 77.0 | 193.7 ± 40.3 | 201.6 ± 38.9 | 189.2 ± 32.5 |
| TCHO | mg/dL | 88.2 ± 10.4 | 86.5 ± 12.7 | 86.7 ± 10.3 | 86.3 ± 7.2 |
| TG | mg/dL | 63.3 ± 19.1 | 64.7 ± 11.5 | 68.0 ± 16.6 | 63.2 ± 6.8 |
| TP | g/dL | 4.89 ± 0.16 | 4.79 ± 0.19 | 4.92 ± 0.24 | 4.89 ± 0.33 |
| ALB | g/dL | 2.23 ± 0.22 | 2.38 ± 0.19 | 2.35 ± 0.21 | 2.45 ± 0.22 |
| A/G ratio | - | 0.85 ± 0.15 | 0.99 ± 0.12* | 0.92 ± 0.11 | 1.03 ± 0.19* |
| BUN | mg/dL | 23.3 ± 9.7 | 19.5 ± 5.1 | 19.6 ± 2.1 | 20.2 ± 2.8 |
| CRE | mg/dL | 0.24 ± 0.03 | 0.24 ± 0.02 | 0.26 ± 0.03 | 0.24 ± 0.02 |
| IP | mg/dL | 7.78 ± 1.67 | 7.44 ± 1.62 | 7.84 ± 1.56 | 7.54 ± 1.27 |
| Ca^2+^ | mg/dL | 7.76 ± 1.22 | 8.23 ± 0.49 | 8.49 ± 0.53 | 8.51 ± 0.37 |
| Na^+^ | mmol/L | 139.3 ± 4.4 | 142.4 ± 3.8 | 142.2 ± 4.2 | 145.9 ± 2.4** |
| K^+^ | mmol/L | 5.36 ± 1.08 | 4.69 ± 0.73 | 4.81 ± 0.75 | 4.64 ± 0.36 |
| Cl^-^ | mmol/L | 112.9 ± 5.1 | 110.5 ± 2.9 | 111.9 ± 2.2 | 114.6 ± 2.6 |
| Number of animals | | 10 | 10 | 10 | 10 |

Abbreviations: AST, Aspartate aminotransferase; ALT, Alanine aminotransferase; ALP, Alkaline phosphatase; CPK, Creatine phosphokinase; TBIL, Total bilirubin; GLU, Glucose; TCHO, Total cholesterol; TG, Triglyceride; TP, Total protein; ALB, Albumin; A/G ratio, Albumin/globulin ratio; BUN, Blood urea nitrogen; CRE, Creatinine; IP, Inorganic phosphorus

Data are presented as the mean ± S.D., with the number of animals in parentheses.

*/** Significant difference at *P*<0.05/*P*<0.01 levels compared with vehicle control.
