# Supplementary material for: Comprehensive Preclinical Safety Evaluation of Clinical‐Grade hESC‐Derived Multipotent Mesenchymal Stem Cells for Interstitial Cystitis
Source: Stem Cells Int. 2026 Aug 18;2026:5539154. doi: 10.1155/sci/5539154 (PMC13484282; doi:10.1155/sci/5539154)
Supplement: Supplementary file 1 [file SCI-2026-5539154-s001.zip › Supporting Information 9. Table S8.docx]

Table S8. Primer and probe sequence used for qRT-PCR in the biodistribution study

| **Genes** | **Sequences** | |
| --- | --- | --- |
| Human *Alu* | Forward | 5’-TTA GCC GGA CGT AGT GGC-3’ |
|  | Reverse | 5’-GCA ATC TCG GCT CAC TGC AA-3’ |
|  | Probe | 5’-6-FAM-AGC TAC TCG GGA GGC TGA GGC AGG A-3BHQ1-3’ |
| Mouse *GAPDH* | Forward | 5’-TGT GAA CCA CGA GAA ATA TGA-3’ |
|  | Reverse | 5’-TTG TCA TGG ATG ACC TTG GC-3’ |
|  | Probe | 5’-HEX-CTC ACT CAA GAT TGT CAG CAA TGC-BHQ1-3’ |
